# Reprogramming of Strawberry (*Fragaria vesca*) Root Transcriptome in Response to *Phytophthora cactorum*

**DOI:** 10.1371/journal.pone.0161078

**Published:** 2016-08-12

**Authors:** Anna Toljamo, Daniel Blande, Sirpa Kärenlampi, Harri Kokko

**Affiliations:** Department of Environmental and Biological Sciences, University of Eastern Finland, Kuopio, Finland; Agriculture and Agri-Food Canada, CANADA

## Abstract

Crown rot (*Phytophthora cactorum*) causes significant economic losses in strawberry production. The best control strategy would be to use resistant cultivars, but polygenically inherited resistance makes the breeding of the garden strawberry (*Fragaria* × *ananassa*) challenging. The diploid wild strawberry *Fragaria vesca* Hawaii 4 genotype was shown previously to have resistance against crown rot. To explore the resistance mechanisms, we inoculated the roots of Hawaii 4 with *P*. *cactorum* in a novel *in vitro* hydroponic system to minimize interference caused by other microbes. Major reprogramming of the root transcriptome occurred, involving 30% of the genes. The surveillance system of the plant shifted from the development mode to the defense mode. Furthermore, the immune responses as well as many genes involved in the biosynthesis of the defense hormones jasmonic acid, ethylene and salicylic acid were up-regulated. Several major allergen-like genes encoding PR-10 proteins were highly expressed in the inoculated plants, suggesting that they also have a crucial role in the defense responses against *P*. *cactorum*. Additionally, flavonoids and terpenoids may be of vital importance, as several genes involved in their biosynthesis were up-regulated. The cell wall biosynthesis and developmental processes were down-regulated, possibly as a result of the down-regulation of the key genes involved in the biosynthesis of growth-promoting hormones brassinosteroids and auxin. Of particular interest was the expression of potential resistance genes in the recently identified *P*. *cactorum* resistance locus *RPc-1*. These new findings help to target the breeding efforts aiming at more resistant strawberry cultivars.

## Introduction

Strawberry crown rot caused by the hemibiotrophic oomycete *Phytophthora cactorum* hampers strawberry production in many parts of the world. It causes stunting and wilting of the plants and spoils the fruits by causing leather rot [1]. The wilting symptoms first appear in the young leaves and as the disease progresses the whole plant may collapse. The most characteristic symptom is brown necrosis in the vascular tissue of the crown. As *P*. *cactorum* is a soil-borne pathogen and forms sexual oospores that may survive many years in the soil, the removal of the disease from the infested fields is difficult. The cultivation practices promoting soil drainage may help in managing the disease. In addition, fosetyl-Al, phosphite-based products or elicitors could provide some protection against crown rot [2]. However, the most effective method to control the disease is the use of resistant cultivars.

Strawberry displays resistance against most *P*. *cactorum* strains, and only specialized *P*. *cactorum* isolates have shown potential to cause crown rot [3,4]. On the other hand, many of the widely used strawberry cultivars are susceptible to specialized *P*. *cactorum* crown rot isolates, and only a few cultivars display resistance quite consistently [5,6]. Shaw et al. have suggested that resistance against crown rot pathotype is a polygenic trait [7,8]. Besides the genetic background, the physiological status of the plant plays a role: cold-stored and/or wounded plants are particularly prone to the disease, and young plants tend to be more susceptible than the older ones [9]. Eikemo et al. tested 60 diploid *Fragaria* sp. accessions for their resistance/susceptibility to *P*. *cactorum* [10]. The accessions varied from resistant to highly susceptible, but a majority was categorized as resistant or moderately resistant. Recently, Davik et al. analyzed the F_2_ population of a cross between crown rot resistant and susceptible *F*. *vesca* genotypes (Bukammen and Haugastøl 3, respectively) [11]. They were able to identify a single major gene locus (named *RPc-1*, *Resistance* to *Phytophthora cactorum 1*), which explained 74.4% of the phenotypic variance. The 3.3 Mb QTL region contained 801 predicted genes, of which 69 may play a role in disease resistance. However, a major effort is still needed to identify those genes actually conferring crown rot resistance.

The defense mechanisms against strawberry pathogens have been extensively reviewed [12]. As in other plants, a two-layered inducible defense system probably functions also in strawberry. Immunity is induced, if the plant recognizes a highly conserved pathogen- or microbe-associated molecular pattern (PAMP or MAMP) with the help of a membrane-localized pattern-recognition-receptor (PRR), or if it perceives a pathogen effector with the help of a resistance (R) protein [13]. The recognition triggers a complex signaling network, leading to the reprogramming of gene expression and to the activation of defense responses, including the synthesis of antimicrobial secondary metabolites and the expression of pathogenesis-related (PR) genes. The magnitude of the defense varies depending on the pathogen, the effector-triggered immunity (ETI) being a stronger and faster response than the PAMP-triggered immunity (PTI). Plant hormones play important roles in the modulation of defense responses. In general, the salicylic acid (SA)-dependent signaling pathway is effective against biotrophic and hemibiotrophic pathogens and is required for the activation of hypersensitive cell death response, whereas the jasmonate (JA)- and ethylene (ET)-mediated defense responses act against necrotrophs [14]. Crosstalk between these two signaling pathways is often antagonistic, and defense responses can be modulated also by other plant hormones [15].

Until now, no large-scale transcriptome analyses on strawberry defense responses against *P*. *cactorum* have been reported. To gain a better understanding of the defense mechanisms, we inoculated *F*. *vesca* plantlets with *P*. *cactorum* zoospores, and sequenced the root transcriptome. The diploid woodland strawberry, *F*. *vesca*, is the wild relative of the octoploid garden strawberry. The accession Hawaii 4 (H4) was chosen as it is quite resistant against crown rot [10], and is the reference accession used in the genomic sequencing project [16]. In order to reduce biological variation between the replicates, micropropagated plant clones were used, and a novel hydroponic *in vitro* culture system was exploited to eliminate interference caused by other microbes (S1 Fig). The samples were collected two days post-inoculation, representing the early infection stage according to Chen et al. [17]. The data provide comprehensive insight into the transcriptional reprogramming that occurs in the roots of *F*. *vesca* upon inoculation.

## Materials and Methods

### Plant material

Micropropagated plants of garden strawberry, *Fragaria* x *ananassa* cv. ‘Senga Sengana’, were purchased from Agrifood Research Finland MTT, Laukaa Research and Elite Plant Station. The seeds of *F*. *vesca*, accession Hawaii 4, were provided by Dr. Timo Hytönen (Department of Agricultural Sciences, University of Helsinki, Finland). The seeds were disinfected in 5% sodium hypochlorite for 2 min, washed in sterile deionized water, and placed on Murashige-Skoog (MS) agar [18]. Two germinated seedlings, named H4.4 and H4.5, and the ‘Senga Sengana’ plants were micropropagated on MS agar supplemented with 6-benzylaminopurine (2,2 mg/l) and indole-3-butyric acid (0,4 mg/l). Temperature of the growth room was 20 ± 2°C and the light/dark cycle was 16/8 h.

To induce the root formation, the micropropagated plants were transplanted to MS agar without hormones. After 4 weeks, when the roots were sufficiently developed, the plants were transferred to aerated hydroponic culture in modified RITA® containers (VITROPIC, Saint-Mathieu-de-Tréviers, France) (S1 Fig). Two plants of each clone (H4.4, H4.5, ‘Senga Sengana’) were placed in each container (six containers in total), and the roots were protected from light by wrapping aluminum foil around the lower part of the container. The plants were grown in modified half-strength Hoagland solution (3 mM KNO_3_, 2 mM Ca(NO_3_)_2_, 1 mM NH_4_H_2_PO_4_, 0.5 mM MgSO_4_, 1 μM KCl, 25 μM H_3_BO_3_, 2 μM ZnSO_4_, 2 μM MnSO_4_, 0.1 μM CuSO_4_, 0.1 μM (NH_4_)_6_Mo_7_O_24_, 20 μM Fe(Na)EDTA, 2 mM MES) for 20 days before the inoculation. The growth room conditions were as described above.

### Oomycete material

The *P*. *cactorum* isolate Pc407 originates from infected rhododendrons and is highly virulent to garden strawberry [19]. The *P*. *cactorum* isolate was maintained on potato dextrose agar (PDA) at the room temperature. To induce sporangia and zoospore production, the isolate was cultured in pea broth and soil extract water. Pea broth was prepared as described by Zentmyer and Chen [20]. Frozen green peas were mixed in a blender in deionized water (100 g/250 ml). After centrifugation, the supernatant was filtered through Whatman® qualitative filter paper (GE Healthcare Bio-Sciences, Pittsburgh, PA, USA), deionized water was added to 500 ml, and the preparation was autoclaved (121°C, 15 min). The soil extract water was prepared according to Hamm & Hansen [21]. The soil (Puutarhamulta, Kekkilä Oy, Vantaa, Finland) was mixed in deionized water (1:1) and kept overnight at room temperature. After centrifugation and filtering through qualitative filter paper, the soil extract water was autoclaved.

The *P*. *cactorum* was grown in the pea broth (10 ml) in Petri dishes for three days at room temperature. To induce sporangia development, the cultures were washed three times with sterilized deionized water, and 10 ml of soil extract water was added. The plates were incubated overnight at room temperature under fluorescent light. The soil extract water was removed and the plates were kept under fluorescent light for another 24 h, after which plenty of sporangia were visible. Sterile deionized water (25 ml, 4°C) was added, and the cultures were chilled for 30 min at 4°C and then returned to room temperature. After 2 h, most of the zoospores were liberated, and the suspensions were decanted to sterile Erlenmeyer flasks. The zoospores were counted (Bürker chamber), and the concentration was adjusted to 5000 zoospores ml^-1^ by adding sterile deionized water.

### Inoculation with *P*. *cactorum* zoospores and sample collection

For the inoculation with *P*. *cactorum* zoospores, the scaffolds, each holding six plants (S1 Fig), were transferred to RITA®-containers (modified for hydroponic growth) containing 390 ml of zoospore suspension or sterile deionized water (negative control). The roots were immersed in the liquid for 30 min, and then returned to half-strength Hoagland solution. All treatments were made in triplicate.

Samples for RNA extraction were collected 2 days after inoculation, as the hyphae were visible on the root surfaces of the inoculated plants (S6 Fig). Three biological replicates were collected, all from different containers. Roots, crown and leaves were excised with scalpel, weighed separately and snap frozen in liquid nitrogen. Samples were stored at—80°C.

### RNA extraction

Roots of H4.4 plants were ground to fine powder in liquid nitrogen using a mortar and pestle, each individual separately. During grinding, 1 ml of SE buffer (0,14 M NaCl, 2 mM KCl, 2 mM KH_2_PO_4_, 8 mM Na_2_HPO_4_*2 H_2_O, 0,05% v/v Tween-20, 2% w/v polyvinylpyrrolidone 40, 0,7% w/v bovine serum albumin) was added to 100 mg of plant material [22,23]. The powder (~ 150 mg) was transferred to microcentrifuge tubes. RNA was extracted with RNeasy Mini Kit (Qiagen, Valencia, CA, USA) according to manufacturer´s instruction. To remove genomic DNA, On-column DNase Digestion was made with RNase-Free DNase Set (Qiagen). The quality and quantity of RNA was determined with Nanodrop® ND-1000 spectrophotometer (NanoDrop Products, Wilmington, DE, USA), and samples with 260/280 and 260/230 absorbance ratios of > 2.0 and > 1.75, respectively, were accepted for sequencing.

### Library preparation and RNA sequencing

RNA sequencing was made to six root samples of Hawaii4.4 (three controls and three inoculated root samples), each representing one individual and collected from a different container. The library construction and sequencing of the samples were performed in Weill Cornell Medical College, Genomics Resources Core Facility (NY, USA). The RNA integrity was verified with Bioanalyzer 2100 (Agilent Technologies, Palo Alto, CA, USA). The libraries were constructed with Illumina Truseq RNA-seq Sample Prep Kit, and the sequencing was made using Illumina HiSeq2000 (Illumina, San Diego, CA, USA). The raw reads are available at the NCBI SRA database (accession numbers SRR3743193, SRR3743194, SRR3743195, SRR3743196, SRR3743197, SRR3743198).

### Data analysis

The raw reads were first trimmed using Trimmomatic (version 0.32) [24]. The adapter sequences were removed, and low quality bases (phred quality score below 3) were deleted from both ends of the reads. The reads were also scanned with 4-base sliding window and cut if the average quality per base dropped below 15. Reads shorter than 36 bases were discarded.

The trimmed reads were mapped against *F*. *vesca* nuclear (version v1.0) and chloroplast genome using STAR RNA-seq aligner (version 2.4.0) [16,25]. Maximum of ten mismatches were allowed, and the minimum and maximum intron lengths were set to 20 and 6000, respectively. Reads that were mapped to coding sequences (CDS) of annotated genes were counted using featureCounts with default settings [26]. Chloroplast genome annotation was released on 12.05.2011 and genome annotation was last updated on 04.03.2015. The unmapped paired-end reads were mapped against expressed sequence tag (EST) sequences of *Phytophthora* species (330 482 sequences downloaded from NCBI EST database) using Bowtie (version 0.12.7) [27].

Differential expression analysis was made using EdgeR [28]. Only the genes with a minimum expression level of one count per million (cpm) in at least three replicates were used. The genes were considered differentially expressed, if the false discovery rate (FDR) was < 0.05 and log_2_ fold change < -1 or > 1.

Gene Ontology (GO) terms were searched for the longest protein sequence of each gene using PANNZER with default settings [29]. GO term enrichment analysis was made using BiNGO plugin in Cytoscape [30]. REVIGO was used to remove redundant terms [31]. Protein domains for receptor-like kinases were searched with Interproscan in Geneious 8.1.5 (http://www.geneious.com)[32]. Separate GO term enrichment analysis was also made for the up- and down-regulated RLK gene sets, using the whole RLK gene set as a reference.

## Results and Discussion

To understand the molecular events resulting from the interaction of *P*. *cactorum* with strawberry roots, we first needed to establish a suitable plant-pathogen pair. Of the *P*. *cactorum* isolate selection in our position, the isolate 407, originating from infected rhododendrons, was known to be highly virulent to garden strawberry [19]. It killed or nearly killed 80% of the inoculated garden strawberry cv. Jonsok plants in pathogenicity tests. When we tested the isolate on another strawberry cv. Senga Sengana known to be one of the most resistant cultivars against crown rot [5,6], it caused severe stunting of the plants (S2 Fig). On the other hand, 407 did not cause significant decrease in the biomass of *F*. *vesca* Hawaii 4.4 either in greenhouse conditions (S2 Fig) or in hydroponic *in vitro* cultures (S3 Fig). We concluded that the *P*. *cactorum* isolate 407 together with *F*. *vesca* genotype Hawaii 4.4 served our purpose, as the isolate was not pathogenic to Hawaii but was pathogenic at least to two garden strawberry cultivars. The responses that would be seen were expected to indicate how *F*. *vesca* protects itself against *P*. *cactorum* and crown rot disease.

### Overview of RNA sequencing results

Illumina sequencing generated 47.36–54.28 million paired-end raw reads (101 bp) per sample (Table 1). Of the read pairs, 94.3% passed the quality filtering, and 61.77–76.18% of those were uniquely mapped to coding DNA sequences (CDS) of the nuclear or chloroplast genome of *F*. *vesca*. In total, 104.79 and 97.24 million reads were uniquely assigned to CDS in water control and inoculated plants, respectively. Of the filtered reads, 4.50%, 8.23% and 1.67% aligned to *Phytophthora* EST sequences in the inoculated samples 1–3, respectively.

**Table 1:** Summary of RNA sequencing results. Controls 1 to 3 refer to water control replicates, inoculated 1 to 3 to replicate plants inoculated with *P*. *cactorum* 407; each replicate represents roots collected from one individual plant.

	Control1	Control2	Control3	Inoculated1	Inoculated2	Inoculated3
Raw reads	47 362 397	53 857 982	52 650 989	51 095 961	54 281 780	48 268 982
Filtered reads	44 737 172	50 780 701	49 622 232	48 143 355	51 128 038	45 564 435
(% of all reads)	94.46	94.29	94.25	94.22	94.19	94.4
Uniquely mapped reads	41 431 462	45 406 557	45 624 736	36 599 168	36 557 345	39 548 772
(% of filtered reads)	92.61	89.42	91.94	76.02	71.5	86.8
Reads assigned to annotated genes	34 082 834	33 195 110	37 515 085	31 698 158	31 582 432	33 963 737
(% of filtered reads)	76.18	65.37	75.6	65.84	61.77	74.54

Genes expressed at a low level were filtered out, leaving 16 793 genes for further analysis (S1 Table). Of these genes, 4576 and 4243 were down- and up-regulated (false discovery rate (FDR) < 0.05) in the inoculated plants compared to water controls, respectively, if no fold-change cut-off values were applied. However, to improve the reliability of the results, more stringent analysis was carried out with cut-off values of log_2_ fold-change < -1 or > 1, resulting in 2993 significantly down-regulated and 2371 up-regulated genes (FDR < 0.05). These more stringent cut-off values were used below. The differences between the groups were mainly quantitative, since only 15 and 10 genes were expressed exclusively in the controls and inoculated plants, respectively.

GO terms were assigned to 13 144 genes (78% of the genes) using PANNZER annotation program (S1 Table). GO term enrichment analysis of the expressed genes revealed 656 and 475 biological processes, 252 and 84 molecular functions and 37 and 85 cellular components significantly enriched (adjusted p-value < 0.05) in the up- and down-regulated gene sets, respectively (S2 Table). The most significantly (adjusted p-value < 1E-15) enriched biological processes are shown in S4 Fig. As one might expect, “response to biotic stimulus” was the most significantly enriched biological process among the gene set up-regulated in the inoculated plants. This confirms that the experimental set-up was successful. Other up-regulated biological processes included many GO terms associated with metabolic/biosynthetic processes, signaling, transport, responses to various stimuli, defense responses and regulation of immune processes (S2 Table). Salicylic acid, jasmonic acid and ethylene biosynthetic/metabolic processes were up-regulated as well.

On the other hand, “cell wall organization or biogenesis” was the most significantly down-regulated biological process. Several GO terms related to polysaccharide metabolism, morphogenesis, development and growth were also down-regulated (S2 Table). Of the hormonal processes, auxin transport and brassinosteroid metabolic/biosynthetic processes were the most significantly down-regulated ones. Down-regulation of growth and development is a well-known phenomenon in pathogen-challenged plants [33]. When the resources are limited, the plant needs to prioritize the defense over development and growth in order to survive. However, constant, unnecessary activation of the defense responses at the expense of development is harmful for the fitness of the plant. In this study, growth repression caused by *P*. *cactorum* inoculation was apparently transient, as there were no significant differences in the biomass between the inoculated and uninoculated plants in the long-term experiments (S2 and S3 Figs). This minuscule effect on growth may be associated to the tight regulation of defense responses. In the next chapters, a closer look is taken to the genes related to the most significantly up- and down-regulated biological processes and the genes that could play important roles in plant immunity.

### Genes expressed in *P*. *cactorum* resistance locus *RPc-1*

Of particular interest was the expression of 69 defense related genes, located in the QTL region associated with *P*. *cactorum* resistance in *F*. *vesca* [11]. Of these genes, 47 were expressed highly enough (at least one cpm in three replicates) to be included in the differential expression analysis (Table 2). Seven of these genes were significantly down-regulated, while 19 were up-regulated upon *P*. *cactorum* inoculation. Many of the up-regulated genes, such as cyclic nucleotide-gated ion channels, calcium-binding protein and kinases are probably involved in signaling events, whereas WRKY transcription factors are important transcriptional regulators of plant defense responses. Phenylalanine ammonia-lyase (PAL) catalyzes the first committed step in the phenylpropanoid pathway, leading to the biosynthesis of various polyphenolic compounds, and is arguably one of the central players in plant defense. However, the most interesting resistance gene candidates are receptor-like protein, receptor kinases and nucleotide-binding site–leucine-rich repeat (NBS-LRR) resistance proteins possibly functioning in pathogen perception. In total, four NBS-LRR protein-encoding genes (101306457, 101297569, 101300750, 101304699) were expressed in the *RPc-1* locus, two of them being significantly down-regulated in the inoculated plants. The most highly expressed NBS-LRR gene was 101297569, which had much higher expression level compared to the other NBS-LRR genes, and can thus be considered as one of the resistance gene candidates in the *RPc-1* locus. Receptor-like kinases (RLK) are discussed in more detail in the next chapter.

**Table 2:** Genes in *P*. *cactorum* resistance locus *RPc-1* expressed in the roots of *F*. *vesca*.

Geneid	Product	Control (cpm)	Inoc. (cpm)	log_2_ FC	FDR
101305393	putative receptor protein kinase ZmPK1	0.00	1.92	9.13	4E-24
101296502	probable WRKY transcription factor 70	0.17	28.30	7.39	8E-38
101297362	probable WRKY transcription factor 70	0.01	2.27	6.94	1E-18
101295534	calcium-binding protein CML42-like	1.49	130.21	6.46	2E-54
101313535	cyclic nucleotide-gated ion channel 1-like	0.17	3.14	4.26	4E-09
101290881	receptor-like protein 12 isoform X1	14.11	161.53	3.52	1E-18
101310048	L-type lectin-domain containing receptor kinase IV.1	25.25	286.54	3.50	6E-36
101311683	probable WRKY transcription factor 33	21.75	154.02	2.82	3E-17
101297653	probable WRKY transcription factor 70	0.36	2.52	2.79	4E-05
101315259	phenylalanine ammonia-lyase 1	89.99	539.49	2.58	7E-16
101305576	MLO-like protein 3	19.50	103.53	2.41	8E-13
101309756	L-type lectin-domain containing receptor kinase S.4-like	28.95	137.88	2.25	3E-20
101305865	MLO-like protein 6	60.25	286.25	2.25	4E-17
101305094	putative receptor protein kinase ZmPK1	0.13	0.56	2.08	6E-04
101292429	cyclic nucleotide-gated ion channel 1-like	47.66	137.03	1.52	2E-04
101312550	ser/thr-protein kinase AtPK2/AtPK19-like	24.89	68.13	1.45	4E-06
101294478	probable ser/thr-protein kinase NAK	52.08	142.24	1.45	1E-08
101309855	putative ser/thr-protein kinase	11.25	29.26	1.38	2E-09
101301595	non-specific lipid-transfer protein 1-like	61.21	142.12	1.22	3E-07
101307520	gamma-interferon-inducible-lysosomal thiol reductase	1.37	2.57	0.91	2E-02
101293913	probable LRR-RLK (At1g06840 ortholog)	4.41	7.62	0.79	1E-02
101291543	CBL-interacting protein kinase 2-like	94.73	160.18	0.76	3E-02
101312949	cyclic nucleotide-gated ion channel 1-like	13.74	22.94	0.74	8E-02
101315445	probable leucine-rich repeat RLK (At5g49770 ortholog)	196.92	292.18	0.57	4E-02
101297067	probable WRKY transcription factor 70	60.45	82.12	0.44	5E-01
101291163	probable LRR-RLK (At3g47570 ortholog)	1.38	1.77	0.36	4E-01
101315161	ser/thr-protein kinase HT1	28.58	36.46	0.35	1E-01
101293218	BTB/POZ domain-containing protein (At3g09030 ortholog)	18.06	22.59	0.32	2E-01
101308191	PHD finger-like domain-containing protein 5B	16.47	18.77	0.19	5E-01
101297170	ABC transporter B family member 1	170.99	183.62	0.10	7E-01
101292428	PHD finger protein ALFIN-LIKE 1-like	114.68	122.32	0.09	7E-01
101299684	BTB/POZ domain-containing protein (At3g08570 ortholog)	23.69	20.89	-0.18	5E-01
101308206	L-type lectin-domain containing receptor kinase VIII.1	21.26	18.03	-0.24	4E-01
101292033	L-type lectin-domain containing receptor kinase VIII.2-like	88.21	72.10	-0.29	2E-01
101306457	putative disease resistance protein RGA3	4.70	3.67	-0.36	3E-01
101297569	putative disease resistance protein RGA3	43.62	32.41	-0.43	5E-02
101293502	ser/thr-protein kinase (At5g01020 ortholog)	27.89	19.05	-0.55	2E-02
101307601	calcium-dependent protein kinase 13	69.97	43.43	-0.69	9E-04
101291255	CBL-interacting ser/thr-protein kinase 14-like	31.23	17.27	-0.86	1E-04
101291075	cyclic nucleotide-gated ion channel 1-like	2.37	1.23	-0.95	2E-02
101292039	probable LRR-RLK (At3g47570 ortholog)	1.73	0.77	-1.16	3E-03
101300750	putative disease resistance protein RGA3	2.33	0.92	-1.35	8E-04
101300640	probable ser/thr-protein kinase (At5g41260 ortholog)	54.90	19.23	-1.51	9E-13
101307025	probable ser/thr-protein kinase WNK5	127.90	32.87	-1.96	4E-13
101304699	putative disease resistance protein (At3g14460 ortholog)	3.22	0.72	-2.15	1E-05
101301793	probable WRKY transcription factor 49	2.09	0.41	-2.34	2E-07
101306748	cyclic nucleotide-gated ion channel 1-like	6.40	0.86	-2.89	1E-12

Mean expression levels (cpm) in the controls and in the inoculated roots, log_2_ fold changes and false discovery rates (FDR).

### Receptor-like kinases

Membrane-bound receptor-like kinases are involved in pathogen recognition and activation of immune responses as well as in the regulation of plant development. Protein domains for annotated receptor-like kinases (RLKs) were searched, and 308 RLKs were classified in nine groups based on their domain structures and similarity to known RLKs (Table 3, S5 Fig). Approximately 66% of these RLKs were differentially expressed upon *P*. *cactorum* attack. Among the up-regulated RLK gene set, several enriched GO terms were related to stress and defense responses, the most significant term being “response to salicylic acid stimulus”. In contrast, most of the enriched GO terms in the down-regulated RLK set were related to the regulation of development and growth, indicating that the surveillance system of the plant was shifted from the development mode to the defense mode.

**Table 3:** Receptor-like kinases expressed in the wild strawberry *F*. *vesca*.

RLK group	Total number of RLKs	Up-regulated RLKs	Down-regulated RLKs
**CRK**	10	6	1
**CrRLK1L**	13	2	4
**G-type-LecRLK**	61	34	7
**L-type-LecRLK**	16	12	0
**LRR-MLD-RLK**	17	8	2
**LRR-RLK**	147	36	58
**MLD-LRR-RLK**	9	2	4
**PR5KL**	17	15	0
**WAK or WAKL**	18	12	1
**Total**	308	127	77

Interestingly, two L-type-lectin-RLKs (101310048, 101309756), two G-type-lectin-RLKs (101305393, 101305094) and one receptor-like protein (RLP) (101290881), significantly up-regulated in the inoculated plants in our study, are located in the QTL region associated with *P*. *cactorum* resistance in *F*. *vesca* [11] (Table 2). Especially, two L-type-lectin-RLKs (101310048, 101309756) were highly expressed and markedly up-regulated in the inoculated plants (Table 2); they were also the most highly expressed L-type-lectin-RLKs in the inoculated roots of *F*. *vesca*. In addition, one gene (101292033) located in the *RPc-1* locus, with typical L-type-lectin receptor domain but lacking kinase domain, was constitutively highly expressed in the roots (Table 2). As several studies have demonstrated the role of L-type-lectin-RLKs in *Phytophthora* resistance [34–36], these genes can be considered as the strongest resistance gene candidates in the *RPc-1* locus. Some G-type-lectin-RLKs are involved in innate immunity as well, although they are better known for their role in the control of self-incompatibility in *Brassicaceae* [37]. For example, NgRLK1 is the interactor of *Phytophthora capsici* elicitin, capsicein, that is able to trigger a hypersensitive response [38]. Up-regulated RLP in the *RPc-1* locus contains a leucine-rich repeat (LRR)-domain in the extracellular part but lacks a cytoplasmic kinase domain. As this gene was highly expressed in the inoculated plants and LRR-RLPs have been implicated in disease resistance in several plants [39], this gene is also a good resistance gene candidate in *F*. *vesca*. To unravel the potential role of these RLKs in the crown rot resistance, mechanistic studies should be carried out.

A closer look at the RLKs revealed a new group of proteins, which often contained wall-associated receptor kinase galacturonan-binding domain (IPR025287) in their extracellular part, although being otherwise more similar to pathogenesis-related 5 (PR5)-like receptor kinase-related (PTHR24420:SF561) subfamily in the PANTHER database. Over 80% of the members of this RLK group were up-regulated in *F*. *vesca* roots after *P*. *cactorum* inoculation (Table 3), suggesting an important role in defense responses. Wall-associated kinases (WAK)/WAK-like (WAKL) may serve as sensors monitoring cell wall integrity, since they are directly cross-linked to cell wall pectins, but are also able to bind shorter pectin fragments released from the cell wall in pathogen attacks and activate defense responses [40]. We also identified an additional RLK group that contained LRR-domain followed by malectin-like carbohydrate-binding domain (IPR024788). Some of the members of this group showed similarity to *Arabidopsis* LysM RLK1-interacting kinase 1, which regulates negatively the innate immunity triggered by chitin and flg22, and positively JA and ET signaling pathways [41]. Of the other RLK groups, the members of which were most often up-regulated, cysteine-rich receptor kinases (CRKs) may perceive extracellular ROS signals and activate intracellular signaling in response to abiotic or biotic stress [42].

Three RLK groups, in which the members were more often down-regulated than up-regulated, were LRR-RLKs, *Catharanthus roseus* RLK1-like kinases (CrRLK1Ls) and MLD-LRR-RLKs (Table 3). CrRLK1Ls regulate cell elongation and developmental processes in plants [43], and many of the down-regulated LRR-RLKs seem to control plant development as well. These data are in line with the results of GO term enrichment analysis indicating the down-regulation of developmental processes upon *P*. *cactorum* inoculation (S4 Fig). Examples of MLD-LRR-RLKs include symbioses receptor-like kinase (SYMRK) required for symbiotic plant-pathogen-interactions [44,45], and impaired oomycete susceptibility 1 (IOS1) that contributes to downy mildew disease development in *Arabidopsis* [46]. As some of the MLD-LRR-RLKs may function as susceptibility factors, it may be beneficial to down-regulate their expression upon pathogen attack.

### Phenylalanine ammonia-lyase genes and positive regulators of SA signaling

The SA-related biological processes were up-regulated in *F*. *vesca* upon *P*. *cactorum* inoculation. In SA-rich plants such as poplar, rice and potato, SA synthesis mainly takes place through phenylalanine ammonia-lyase (PAL)-dependent phenylpropanoid pathway [47]. As strawberry leaves have high basal levels of SA [48], it seems probable that phenylpropanoid pathway is the main SA biosynthesis pathway also in *Fragaria* spp. Of the two PAL-encoding genes expressed in our transcriptome, one (101315259) is located in the *P*. *cactorum* resistance locus in *F*. *vesca* genome [11].

In *Arabidopsis*, SA is mostly synthesized through isochorismate synthase 1 (ICS1) [49], which has not been identified in *F*. *vesca*. However, some other gene homologs known to be involved in SA signaling in *Arabidopsis* were up-regulated in inoculated *F*. *vesca* roots. These included enhanced disease susceptibility 1 (EDS1) and phytoalexin-deficient 4 (PAD4) that function upstream of SA synthesis and potentiate SA signaling in a positive feedback loop [50]. EDS1 is able to form heterodimers with PAD4 and also with senescence-associated gene101 (SAG101) that has been implicated in plant immunity [51]. In our study, three SAG101-genes were up-regulated in the inoculated plants. In addition, two AGD2-like defense response protein 1 (ALD1) genes and two flavin-dependent monooxygenase 1 (FMO1) genes were up-regulated. FMO1 is required for systemic accumulation of SA in systemic acquired resistance (SAR) along with ALD1, which is responsible for the synthesis of pipecolic acid, a long distance signal for SA [52]. The results suggest that systemic responses are activated in the inoculated *F*. *vesca* roots.

### JA and ET biosynthesis and metabolism

The biosynthetic and metabolic processes of JA and ET were significantly enriched among the gene set up-regulated in the *P*. *cactorum* inoculated *F*. *vesca* roots. Two 1-aminocyclopropane-1-carboxylate (ACC) synthase-like (ACS) genes and at least two ACC oxidase-like (ACO) genes were up-regulated in the ET biosynthetic pathway. Also three ethylene response factor 1 (ERF1) genes were up-regulated. ERF1 is known to activate transcription of several PR genes in response to ET and JA and is an important transcription factor in plant defense [53]. Also the negative regulators of ET signaling were up-regulated upon *P*. *cactorum* attack: reversion-to-ethylene sensitivity 1 (RTE1) and two ET receptors (ethylene receptor 2 (ETR2) and ethylene response sensor 1 (ERS1)). These receptors serve as negative regulators of ET signaling, ensuring that the responses are down-regulated as soon as the ET level is diminished [54]. As the prolonged up-regulation of immune responses can be detrimental, this feedback regulation system is probably crucial for the plant.

Most genes in key steps of JA biosynthesis were up-regulated upon *P*. *cactorum* attack: three LOX-like genes, two allene oxide synthase-like (AOS) genes, two allene oxide cyclase-like (AOC) genes, and three 12-oxophytodienoate reductase 3-like genes (OPR3). Activation of negative regulation was also observed. Four negative regulators of JA-responsive genes, jasmonate ZIM-domain (JAZ) genes, were significantly up-regulated, whereas the Coronatine-insensitive protein 1 (COI1)–encoding transcripts were reduced (log_2_ FC -0.955, FDR 4.12E-05). This could cause desensitization to JA-Ile, since COI1 functions as JA-Ile receptor, and forms SCF^COI1^ E3 ubiquitin ligase complexes with other proteins to recruit JAZ repressors for ubiquitination and degradation [55]. Similar responses have been observed in potato cultivar Ackersegen that displays quantitative resistance against *Phytophthora infestans*: concentrated *P*. *infestans* culture filtrate caused up-regulation of genes implicated in SA-related defense responses and JA biosynthesis, but also up-regulation of JAZ1 and down-regulation of COI1 [56]. This suggests that the seemingly contradictory responses could represent a highly functional strategy against *Phytophthora* pathogens.

### WRKY transcription factors potentially involved in SA/JA crosstalk

In total, 21 genes encoding WRKY transcription factors were significantly up-regulated in response to *P*. *cactorum* inoculation. Four of these (101296502, 101297653, 101297362, 101311683) are located in the QTL region associated with *P*. *cactorum* resistance in *F*. *vesca* [11]. In *Arabidopsis*, several of these up-regulated WRKY transcription factors, including WRKY70, promote SA-related defense responses and suppress JA responses [57]. On the other hand, two genes showing similarity to *Arabidopsis* WRKY33, and two probable WRKY40 genes that function as negative regulators of SA/EDS1-signaling pathway and positive regulators of JA signaling were also up-regulated [58,59]. The results suggest that hormone crosstalk in *F*. *vesca* is highly complex and the defense responses are under strict control.

### PR-10 proteins and flavonoid biosynthesis

In total, 18 genes with similarity to Mal d 1, Pru ar 1 or Pru av 1, major allergens of the Rosaceae family, were significantly up-regulated by *P*. *cactorum*. Four of these were among the 10 most highly expressed genes in the *P*. *cactorum* inoculated *F*. *vesca*. All of these proteins belong to the PR-10 pathogenesis-related protein family induced by various stresses [60]. Previous studies have shown that levels of PR-10 transcripts/proteins are elevated in *Fragaria* species challenged with pathogens such as *Colletotrichum acutatum*, *Fusarium oxysporum* f. sp. *fragariae*, *Botrytis cinerea* and *Podosphaera aphanis* [61–65]. The importance of these proteins in disease resistance is supported by the fact that their expression patterns differ between resistant and susceptible plants: some PR-10 proteins are expressed exclusively in the resistant genotype, or their up-regulation is faster or stronger compared to the susceptible genotype.

**Fig 1:**
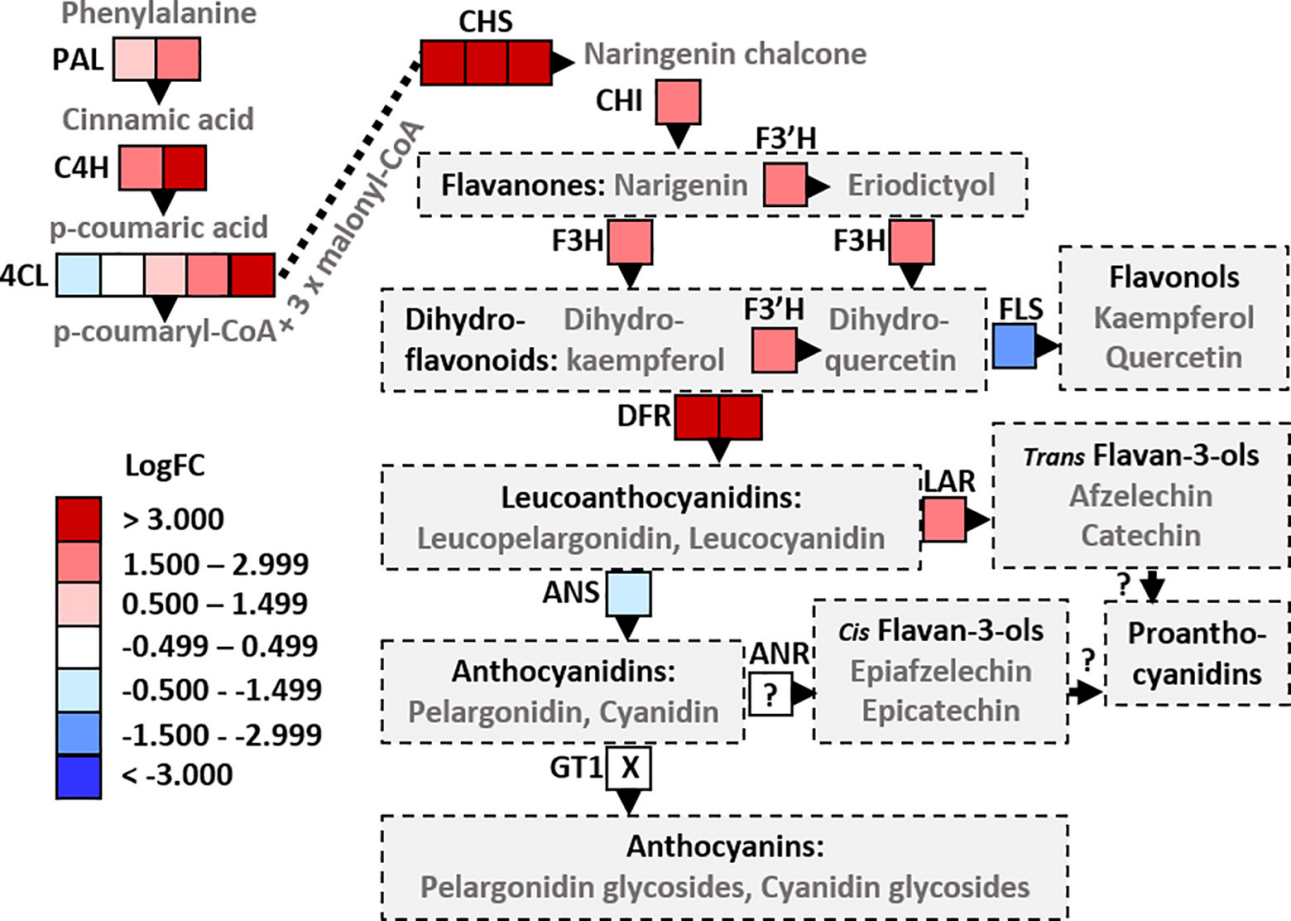
Several genes involved in flavonoid biosynthesis were up-regulated in *F*. *vesca* upon *P*. *cactorum* inoculation. PAL, phenylalanine ammonia-lyase; C4H, cinnamic acid 4-hydroxylase; 4CL, 4-coumarate:coenzyme A ligase; CHS, chalcone synthase; CHI, chalcone isomerase; F3H, flavonoid 3-hydroxylase; F3’H, flavanone 3’-hydroxylase; DFR, dihydroflavanol 4-reductase; ANS, anthocyanidin synthase; GT, glycosyltransferase; FLS, flavonol synthase; LAR, leucoanthocyanidin reductase. GT1 was expressed at low level and it was excluded from the analysis in the filtering step. The expression level of ANR is not known, since it is not included in the annotation version used in this study.

The PR-10 proteins can bind biologically important ligands in their hydrophobic cavity, including fatty acids, cytokinins, sterols and flavonoids [66]. For example, the strawberry Fra a allergens bind metabolic intermediates of flavonoid biosynthesis and thereby control their biosynthesis [67,68]. Thus, it is possible that the up-regulation of the PR-10 proteins in *P*. *cactorum* inoculated *F*. *vesca* is related to the enhanced synthesis of secondary metabolites. In accordance with this, the data suggest that the biosynthesis of phenylpropanoids and flavonoids were up-regulated (Fig 1). In particular, the biosynthesis of flavan-3-ols, such as catechin, was probably increased, since leucoanthocyanidin reductase-like gene was up-regulated, but flavanol synthase and anthocyanidin synthase were significantly and nearly significantly (log_2_ FC: -0.92, FDR: 0.008) down-regulated, respectively. Previous studies have shown that catechin is one of the most abundant flavonoids in strawberry fruits and leaves [69–71]. Catechin seems to be involved in resistance against *Alternaria alternata* and *Botrytis cinerea* in the leaves and fruits of strawberry, respectively [72,73]. We thus suggest that catechin or other flavan-3-ol derived compounds play a role in strawberry defense responses against *P*. *cactorum*.

### Terpenoid biosynthesis

**Fig 2:**
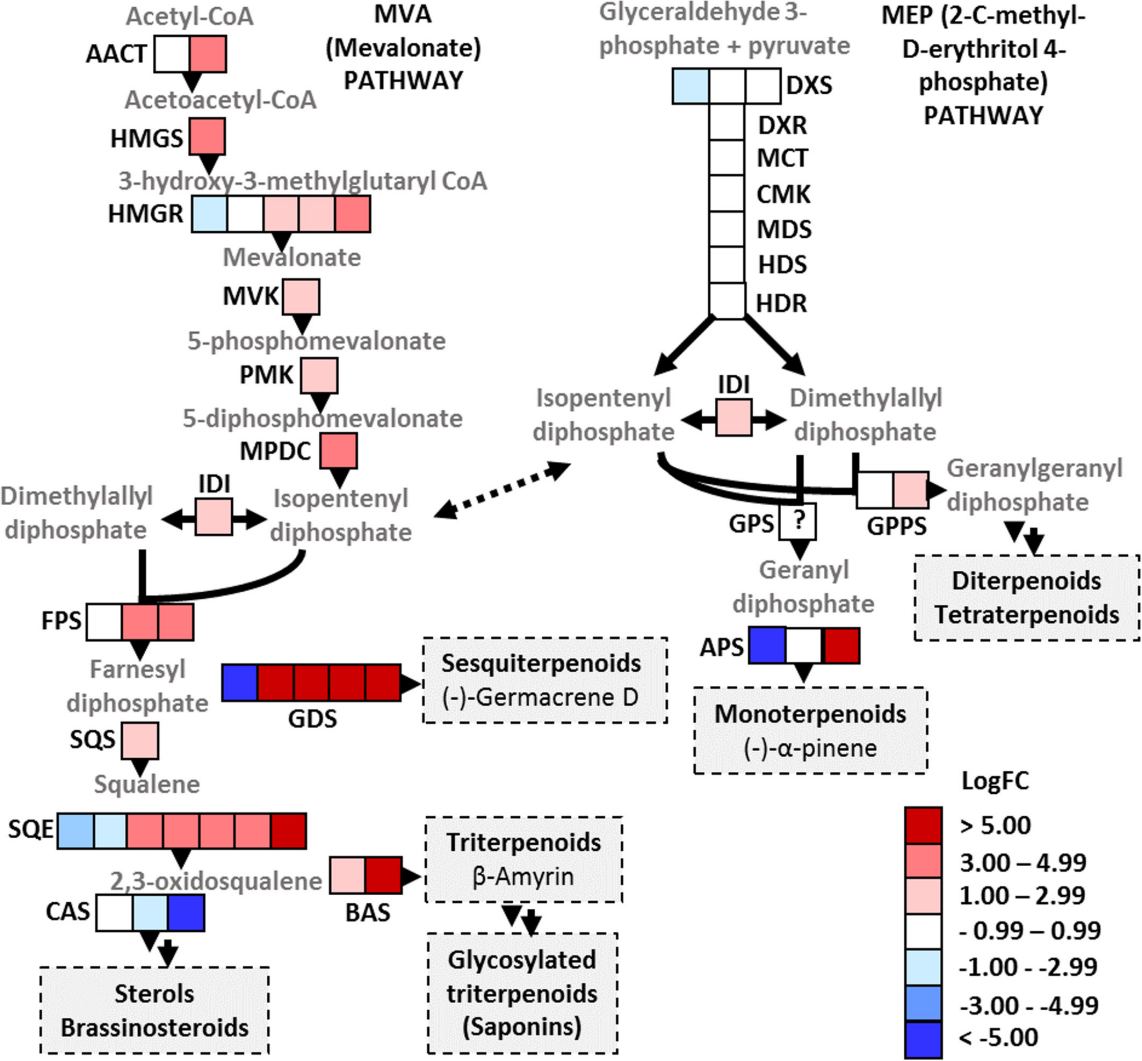
*P*. *cactorum* inoculation changes isoprenoid metabolism in *F*. *vesca* roots. Several genes involved in MVA pathway were up-regulated, whereas none of the MEP pathway genes were up-regulated. Products of the MVA pathway appear to be targeted to sesquiterpenoid and triterpenoid biosynthesis rather than to sterol biosynthesis. AACT, acetoacetyl-CoA thiolase; HMGS, 3-hydroxy-3-methylglutaryl-CoA synthase; HMGR, 3-hydroxy-3-methylglutaryl-CoA reductase; MVK, mevalonate kinase; PMK, phosphomevalonate kinase; MPDC, diphosphomevalonate decarboxylase; IDI, isopentenyl-diphosphate Delta-isomerase; DXS, 1-deoxy-D-xylulose-5-phosphate synthase; DXR, 1-deoxy-D-xylulose 5-phosphate reductoisomerase; MCT, 2-C-methyl-D-erythritol 4-phosphate cytidyltransferase; CMK, 4-(cytidine 5’-diphospho)-2-C-methyl-D-erythritol kinase; MDS, 2-C-methyl-D-erythritol 2,4-cyclodiphosphate synthase; HDS, 1-hydroxy-2-methyl-2-butenyl 4-diphosphate synthase; HDR, 1-hydroxy-2-methyl-2-butenyl 4-diphosphate reductase; FPS, farnesyl diphosphate synthase; GPPS, geranyl diphosphate synthase; GGPPS, geranylgeranyl diphosphate synthase; SQS, squalene synthase; SQE, squalene epoxidase; GDS, (-)-germacrene D synthase-like; BAS, beta-amyrin synthase-like; CAS, cycloartenol synthase -like; APS, (-)-alpha-pinene synthase-like.

Several genes involved in terpenoid biosynthesis according to KEGG (Kyoto encyclopedia of genes and genomes) PATHWAY database [74] were up-regulated in *P*. *cactorum* inoculated *F*. *vesca* (Fig 2). This includes eight genes that catalyze the steps from acetyl-CoA to isopentenyl pyrophosphate (IPP) in the mevalonate (MVA) pathway. In contrast, none of the genes involved in the methylerythritol phosphate (MEP) pathway were up-regulated. The MVA pathway supplies precursors for the biosynthesis of sesquiterpenoids and triterpenoids and functions in the cytosol/endoplasmic reticulum/peroxisomes, whereas the MEP pathway is located in the plastids and gives rise to monoterpenoids, diterpenoids and tetraterpenoids [75]. Even though the MEP pathway was not up-regulated, there could be some changes in the monoterpenoid biosynthesis, as an (-)-alpha-pinene synthase-like gene was strongly up-regulated.

Genes involved in sesquiterpenoid and triterpenoid synthesis were strongly up-regulated. Indeed, the most highly expressed gene in the inoculated plants encodes (-)-germacrene D synthase-like (GDS) protein included in sesquiterpenoid biosynthesis (S1 Table). Three other GDS genes were also significantly up-regulated. Transcript levels of squalene synthase, five squalene epoxidases and two beta-amyrin synthase-like genes were significantly higher in the inoculated roots, suggesting that triterpenoid biosynthesis is increased upon *P*. *cactorum* inoculation. Alpha/beta-amyrin synthase gene, along with 3-hydroxy-3-methylglutaryl coenzyme-A synthase gene, were induced also in the fruits of garden strawberry 24 h after *Colletotrichum acutatum* inoculation [62]. Moreover, Hirai et al. have identified three antifungal triterpenoids from the inoculated and wounded fruits of strawberry cultivar that displays resistance against *Colletotrichum fragariae* [76]. These results suggest that terpenoid biosynthesis is part of the general defense responses in strawberry.

Triterpenoids can be further processed to triterpenoid saponins that consist of triterpenoid backbone with one or more covalently linked sugar moieties [77]. Prior to glycosylation, triterpenoid backbones are often oxygenated by cytochrome P450 enzymes. In our study, ten of the fifteen beta-amyrin 28-oxidase-like genes expressed in *F*. *vesca* were up-regulated, suggesting that triterpenoid saponin biosynthesis is induced in the roots in response to *P*. *cactorum* attack. In a non-targeted metabolite analysis, triterpenoid saponins and glycosylated sesquiterpenoid derivatives have been identified in the strawberry flower [78]. They are enriched in the sepals that protect floral buds from biotic and abiotic stresses, supporting an important role in defense. The molecular mechanisms of antimicrobial and antiherbivorous activities of saponins are not completely understood, but many of them are able to perturb the membranes [79].

### Brassinosteroid biosynthesis

In contrast to terpenoid biosynthesis, some of the genes catalyzing the biosynthesis of steroids and brassinosteroids (BR) were down-regulated in *F*. *vesca* upon *P*. *cactorum* inoculation. This includes two genes encoding cycloartenol synthases (Fig 2), a gene encoding homolog of *Arabidopsis* DWF1 (DWARF1) and a gene encoding CYP85A2/BR6OX2 (BRASSINOSTEROID-6-OXIDASE 2). Cycloartenol synthases catalyze the first step in the pathway leading to campesterol and BR, i.e. the conversion of (S)-2,3-oxidosqualene to cycloartenol. DWF1 catalyzes the conversion of 24-methylenecholesterol to campesterol and CYP85A2/BR6OX2 catalyzes the last, rate-limiting step [80] in BR biosynthesis. We suggest that upon *P*. *cactorum* inoculation squalene is directed to triterpenoid biosynthesis rather than to BRs or other steroids. Furthermore, a homologue of *Arabidopsis* BR receptor, BRI1 (BRASSINOSTEROID INSENSITIVE1), was significantly down-regulated, presumably causing desensitization to BRs.

Down-regulation of BR biosynthesis and reduction of BR responsiveness is in agreement with the regulatory role of BRs in growth-immunity tradeoffs [81]. Even though BRs can enhance pathogen resistance in some cases, several reports indicate negative effects on immunity, in particular on the activation of PAMP-triggered immunity [82,83]. BR treatment also blocks benzothiadiazole (BTH)-induced resistance in rice [84]. It makes sense that BR synthesis would be diminished and BR responsiveness reduced in resistant *F*. *vesca* when inoculated with *P*. *cactorum*, as BR signaling could otherwise override defense signaling. In addition, it would be beneficial for *F*. *vesca* to channel isoprenoid metabolites to terpenoid biosynthesis rather than to BR synthesis.

### Auxin biosynthesis and transport

The transcriptome results suggest a decrease in auxin biosynthesis in the inoculated plants. All four YUCCA genes of *F*. *vesca* (YUC1, YUC3, YUC5, YUC7) [85] expressed in the roots of the *F*. *vesca* H4.4 genotype, were significantly down-regulated. The YUC flavin monoxygenase-like proteins catalyze the rate-limiting step in the indole-3-pyruvic acid (IPA) pathway, which has been confirmed as the main auxin biosynthesis route in *Arabidopsis* [86,87]. According to GO term enrichment analysis, auxin transport was apparently down-regulated as well, and the expression levels of at least seven homologues of genes implicated in auxin transport in *Arabidopsis* were reduced in the inoculated *F*. *vesca* roots.

Auxin is an important regulator of development, and its impact on immunity can be negative or positive [88]. In general, auxin signaling seems to improve resistance against necrotrophs but enhance susceptibility against biotrophic pathogens, apparently because of the antagonistic relationship between SA and auxin. It has been reported that a mutation in WALLS ARE THIN1 (WAT1) gene, encoding a vacuolar auxin transporter, reduces auxin level and increases SA level in *Arabidopsis* roots, which confers resistance to several vascular pathogens [89,90]. As WAT1 gene was down-regulated in the inoculated *F*. *vesca*, it could play a role also in the defense responses against *P*. *cactorum*. Auxin may enhance the susceptibility also by other mechanisms, e.g. by modulating cell wall properties [91,92]. On the other hand, it has been shown that suppression of auxin response renders *Arabidopsis* more susceptible to *Phytophthora cinnamomi* [93]. Because of these contradictory findings it is difficult to conclude whether the changes in auxin-related processes promote or impede immunity in *F*. *vesca*.

### Cell wall biosynthesis

Several GO terms related to cell wall biosynthesis, development and growth were enriched in the gene set down-regulated upon *P*. *cactorum* inoculation. Of the transcription factors regulating cell wall biosynthesis, two MYB46, VASCULAR-RELATED NAC-DOMAIN 4 (VND4) and SECONDARY WALL-ASSOCIATED NAC DOMAIN 2 (SND2) genes were down-regulated in the *P*. *cactorum* inoculated plants. In *Arabidopsis*, MYB46 acts as a master switch activating genes involved in the secondary cell wall synthesis [94], VNDs (VASCULAR-RELATED NAC-DOMAIN) are central activators of the secondary wall synthesis in the vessels [95], and SND2 regulates genes involved in cellulose and hemicellulose biosynthesis and lignin polymerization in fibers [96].

Of the cell wall biosynthetic genes, six out of eight cellulose synthase A catalytic subunit-like genes were down-regulated in the inoculated *F*. *vesca* (Table 4). Another group of genes systematically down-regulated was Fasciclin-like arabinogalactan proteins (FLAs) (Table 4), which are suggested to have a role in cellulose deposition in the secondary cell wall [97]. Of the genes encoding Trichome birefringence-like (TBL) proteins, over 50% were down-regulated (Table 4). The functions of TBLs are not well known, but some of them appear to contribute to the acetylation of cell wall polymers [98]. The observed down-regulation of several cell wall synthesis genes may promote the activation of defense responses, as mutant plants with defects in the cell wall synthesis sometimes display changes in hormone homeostasis and enhanced resistance to pathogens [99]. The only gene group involved in the cell wall synthesis that showed strong up-regulation in the inoculated plants, was the lignin-forming anionic peroxidase-like genes (Table 4), suggesting that the plant attempts to compensate reduced cellulose synthesis by enhancing lignin deposition. This is in agreement with the finding that reduced cellulose synthesis leads to ectopic lignin synthesis in *Arabidopsis* [100].

**Table 4:** Effect of *P*. *cactorum* inoculation of *F*. *vesca* roots on the expression of genes involved in cell wall synthesis.

	Number of genes	Up-regulated	Down-regulated
**Cellulose synthase A catalytic subunit**	8	0	6
**Cellulose synthase -like protein**	12	4	1
**COBRA-like protein**	7	1	3
**Expansins/Expansin-like**	21	3	13
**Fasciclin-like protein**	13	0	9
**Laccase**	33	9	15
**Lignin-forming anionic peroxidase**	3	3	0
**Trichome birefringence-like protein**	34	2	19

## Conclusion

This RNA-seq analysis provides comprehensive insight into the molecular survival mechanisms triggered in *F*. *vesca* roots upon *P*. *cactorum* attack, and emphasizes the balance between development and defense. Accordingly, cell wall synthesis is down-regulated, RLK-based surveillance system and biosynthesis pathways of hormones are reprogrammed and expression of genes involved in the biosynthesis of potential defense compounds is enhanced. Interesting changes were also seen in the recently identified *P*. *cactorum* resistance locus *RPc-1*. Particularly, we want to highlight NBS-LRR resistance gene (101297569), which was the most highly expressed NBS-LRR gene in this locus, and the L-type-lectin-RLKs that were highly expressed and strongly up-regulated in the inoculated roots. Because L-type-lectin-RLKs are known to play important roles in *Phytophthora*-plant interactions, these RLKs are among the most interesting resistance gene candidates in *F*. *vesca* and their function should be further characterized. In addition, metabolomic studies should be carried out to confirm the changes observed in the biosynthesis pathways.

## Supporting Information

S1 Fig *In vitro* infection system. The plants were grown and inoculated in the aerated hydroponic cultures in modified RITA® containers (VITROPIC, Saint-Mathieu-de-Tréviers, France).
(TIF)

S2 Fig Above-ground biomass (g) of control plants and *P*. *cactorum* 407 -inoculated plants of *F*. *vesca* genotype Hawaii 4.4 and garden strawberry cv. Senga Sengana. Micropropagated plants were grown in pots in peat-sand mixture (3:1) for three weeks and inoculated four times with 5 ml of zoospore suspension 21, 29, 32, and 43 days after potting. The zoospore concentrations were 47 500, 130 000, 49 000, 41 000 zoospores /ml, respectively. Biomasses were measured 57 days after first inoculation. *P*. *cactorum* isolate 407 did not reduce the growth of Hawaii 4.4 genotype, but inoculated Senga Sengana plants were severely stunted compared to the controls (p = 0.000001). The means are derived from ten replicates.
(TIF)

S3 Fig Biomass of control plants and *P*. *cactorum* 407 -inoculated plants of *F*. *vesca* Hawaii 4.4 genotype. Micropropagated plants were grown in hydroponic cultures for 32 days, and then inoculated by dipping the roots in zoospore suspension (500 zoospores/ml) for two hours. Biomasses were measured 8 weeks after inoculation. *P*. *cactorum* inoculation did not cause significant reduction in biomass of Hawaii 4.4 plants.
(TIF)

S4 Fig The most significantly enriched biological processes in the *P*. *cactorum* -inoculated *F*. *vesca* roots: (A) up-regulated, (B) down-regulated (adjusted p-value < 1E-15). The most redundant GO terms based on REVIGO analysis were removed to improve clarity. Cutoff value for similarity of 0.7 was used for up-regulated and of 0.9 for the down-regulated processes. The intensity of the color indicates the degree of significance and the node size is proportional to the number of the genes assigned to each GO term in the tested gene set.
(TIF)

S5 Fig Typical domain structures of RLKs. (TIF)

S6 Fig *P*. *cactorum* hyphae on the root surfaces of the inoculated plant two days after inoculation. (TIF)

S1 Table Differential expression analysis. Expression levels (cpm) of *F*. *vesca* genes in the controls and in the inoculated roots, log_2_ fold changes, false discovery rates (FDR) and GO terms. Expression data of all genes are presented on the first spreadsheet of the excel table and the discussed gene groups are presented on separate spreadsheets.
(XLSX)

S2 Table GO term enrichment analysis. Enriched GO terms (biological processes, molecular functions and cellular components) in the up-regulated and down-regulated gene set.
(XLSX)

## References

[1] Maas JL. Compendium of strawberry diseases 2nd ed. St. Paul: American Phytopathological Society (APS Press); 1998.

[2] Eikemo H, Stensvand A, Tronsmo A. Induced resistance as a possible means to control diseases of strawberry caused by *Phytophthora* spp. Plant Dis. 2003;87: 345–350. 10.1094/PDIS.2003.87.4.345 30831827

[3] Bhat R, Colowit P, Tai T, Aradhya M, Browne G. Genetic and pathogenic variation in *Phytophthora cactorum* affecting fruit and nut crops in California. Plant Dis. 2006;90: 161–169. 10.1094/PD-90-0161 30786407

[4] Eikemo H, Klemsdal SS, Riisberg I, Bonants P, Stensvand A, Tronsmo AM. Genetic variation between *Phytophthora cactorum* isolates differing in their ability to cause crown rot in strawberry. Mycol Res. 2004;108: 317–324. 15185982 10.1017/s0953756204009244

[5] Parikka P. Susceptibility of strawberry varieties to crown rot (*Phytophthora cactorum*) in greenhouse tests. Acta Hort. 2003;626: 183–189.

[6] Eikemo H, Stensvand A, Davik J, Tronsmo A. Resistance to crown rot (*Phytophthora cactorum*) in strawberry cultivars and in offspring from crosses between cultivars differing in susceptibility to the disease. Ann Appl Biol. 2003;142: 83–89.

[7] Shaw DV, Hansen J, Browne GT. Genotypic variation for resistance to *Phytophthora cactorum* in a California strawberry breeding population. J Am Soc Hort Sci. 2006;131: 687–690.

[8] Shaw D, Hansen J, Browne G, Shaw S. Components of genetic variation for resistance of strawberry to *Phytophthora cactorum* estimated using segregating seedling populations and their parent genotypes. Plant Pathol. 2008;57: 210–215.

[9] Eikemo H, Stensvand A, Tronsmo A. Evaluation of methods of screening strawberry cultivars for resistance to crown rot caused by *Phytophthora cactorum*. Ann Appl Biol. 2000;137: 237–244.

[10] Eikemo H, Brurberg MB, Davik J. Resistance to *Phytophthora cactorum* in diploid *Fragaria* species. HortScience. 2010;45: 193–197.

[11] Davik J, Eikemo H, Brurberg MB, Sargent DJ. Mapping of the *RPc-1* locus for *Phytophthora cactorum* resistance in *Fragaria vesca*. Mol Breed. 2015;35: 1–11.

[12] Amil-Ruiz F, Blanco-Portales R, Muñoz-Blanco J, Caballero JL. The strawberry plant defense mechanism: a molecular review. Plant Cell Physiol. 2011;52: 1873–1903. 10.1093/pcp/pcr136 21984602

[13] Jones JD, Dangl JL. The plant immune system. Nature. 2006;444: 323–329. 17108957 10.1038/nature05286

[14] Glazebrook J. Contrasting mechanisms of defense against biotrophic and necrotrophic pathogens. Annu Rev Phytopathol. 2005;43: 205–227. 16078883 10.1146/annurev.phyto.43.040204.135923

[15] Robert-Seilaniantz A, Grant M, Jones JD. Hormone crosstalk in plant disease and defense: more than just jasmonate-salicylate antagonism. Annu Rev Phytopathol. 2011;49: 317–343. 10.1146/annurev-phyto-073009-114447 21663438

[16] Shulaev V, Sargent DJ, Crowhurst RN, Mockler TC, Folkerts O, Delcher AL, et al The genome of woodland strawberry (*Fragaria vesca*). Nat Genet. 2011;43: 109–116. 10.1038/ng.740 21186353 PMC3326587

[17] Chen X, Klemsdal SS, Brurberg MB. Identification and analysis of *Phytophthora cactorum* genes up-regulated during cyst germination and strawberry infection. Curr Genet. 2011;57: 297–315. 10.1007/s00294-011-0348-0 21698431

[18] Murashige T, Skoog F. A revised medium for rapid growth and bio assays with tobacco tissue cultures. Physiol Plantarum. 1962;15: 473–497.

[19] Rytkönen A, Lilja A, Vercauteren A, Sirkiä S, Parikka P, Soukainen M, et al Identity and potential pathogenicity of *Phytophthora* species found on symptomatic *Rhododendron* plants in a Finnish nursery. Can J Plant Pathol. 2012;34: 255–267.

[20] Zentmyer G, Chen D. Production of sporangia by *Phytophthora cinnamomi* in pure culture. California Avocado Society, Yearbook. 1969;53: 103–107.

[21] Hamm P, Hansen E. The isolation and identification of Phytophthora species causing damage in bare-root conifer nurseries. Proceedings of the IUFRO Working Party S2 07–09: Diseases and insects in forest nurseries. 1991: 169–179.

[22] van de Heuvel J, Peters D. Improved detection of potato leafroll virus in plant material and in aphids. Phytopathology. 1989;79: 963–967.

[23] Thompson JR, Wetzel S, Klerks M, Vašková D, Schoen C, Špak J, et al Multiplex RT-PCR detection of four aphid-borne strawberry viruses in *Fragaria* spp. in combination with a plant mRNA specific internal control. J Virol Methods. 2003;111: 85–93. 12880923 10.1016/s0166-0934(03)00164-2

[24] Bolger AM, Lohse M, Usadel B. Trimmomatic: a flexible trimmer for Illumina sequence data. Bioinformatics. 2014;30: 2114–2120. 10.1093/bioinformatics/btu170 24695404 PMC4103590

[25] Dobin A, Davis CA, Schlesinger F, Drenkow J, Zaleski C, Jha S, et al STAR: ultrafast universal RNA-seq aligner. Bioinformatics. 2013;29: 15–21. 10.1093/bioinformatics/bts635 23104886 PMC3530905

[26] Liao Y, Smyth GK, Shi W. featureCounts: an efficient general purpose program for assigning sequence reads to genomic features. Bioinformatics. 2014;30: 923–930. 10.1093/bioinformatics/btt656 24227677

[27] Langmead B, Trapnell C, Pop M, Salzberg SL. Ultrafast and memory-efficient alignment of short DNA sequences to the human genome. Genome Biol. 2009;10: 1. 10.1186/gb-2009-10-3-r25 PMC2690996 19261174

[28] Robinson MD, McCarthy DJ, Smyth GK. edgeR: a Bioconductor package for differential expression analysis of digital gene expression data. Bioinformatics. 2010;26: 139–140. 10.1093/bioinformatics/btp616 19910308 PMC2796818

[29] Koskinen P, Törönen P, Nokso-Koivisto J, Holm L. PANNZER: high-throughput functional annotation of uncharacterized proteins in an error-prone environment. Bioinformatics. 2015;31: 1544–1552. 10.1093/bioinformatics/btu851 25653249

[30] Maere S, Heymans K, Kuiper M. BiNGO: a Cytoscape plugin to assess overrepresentation of gene ontology categories in biological networks. Bioinformatics. 2005;21: 3448–3449. 15972284 10.1093/bioinformatics/bti551

[31] Supek F, Bošnjak M, Škunca N, Šmuc T. REVIGO summarizes and visualizes long lists of gene ontology terms. PloS one. 2011;6: e21800 10.1371/journal.pone.0021800 21789182 PMC3138752

[32] Kearse M, Moir R, Wilson A, Stones-Havas S, Cheung M, Sturrock S, et al Geneious Basic: an integrated and extendable desktop software platform for the organization and analysis of sequence data. Bioinformatics. 2012;28: 1647–1649. 10.1093/bioinformatics/bts199 22543367 PMC3371832

[33] Huot B, Yao J, Montgomery BL, He SY. Growth–defense tradeoffs in plants: a balancing act to optimize fitness. Mol Plant. 2014;7: 1267–1287. 10.1093/mp/ssu049 24777989 PMC4168297

[34] Bouwmeester K, de Sain M, Weide R, Gouget A, Klamer S, Canut H, et al The lectin receptor kinase LecRK-I.9 is a novel *Phytophthora* resistance component and a potential host target for a RXLR effector. PLoS Pathog. 2011;7: e1001327 10.1371/journal.ppat.1001327 21483488 PMC3068997

[35] Wang Y, Bouwmeester K, Beseh P, Shan W, Govers F. Phenotypic analyses of *Arabidopsis* T-DNA insertion lines and expression profiling reveal that multiple L-type lectin receptor kinases are involved in plant immunity. Mol Plant-Microbe Interact. 2014;27: 1390–1402. 10.1094/MPMI-06-14-0191-R 25083911

[36] Wang Y, Weide R, Govers F, Bouwmeester K. L-type lectin receptor kinases in *Nicotiana benthamiana* and tomato and their role in *Phytophthora* resistance. J Exp Bot. 2015;66: 6731–6743. 10.1093/jxb/erv379 26248665 PMC4623685

[37] Nasrallah JB, Nasrallah ME. S-locus receptor kinase signalling. Biochem Soc Trans. 2014;42: 313–319. 10.1042/BST20130222 24646237

[38] Kim Y, Oh J, Kim K, Uhm J, Lee B. Isolation and characterization of *NgRLK1*, a receptor-like kinase of *Nicotiana glutinosa* that interacts with the elicitin of *Phytophthora capsici*. Mol Biol Rep. 2010;37: 717–727. 10.1007/s11033-009-9570-y 19449126 PMC2797858

[39] Zipfel C. Plant pattern-recognition receptors. Trends Immunol. 2014;35: 345–351. 10.1016/j.it.2014.05.004 24946686

[40] Kohorn BD. The state of cell wall pectin monitored by wall associated kinases: A model. Plant Signal Behav. 2015;10: e1035854 10.1080/15592324.2015.1035854 26251881 PMC4622591

[41] Le MH, Cao Y, Zhang X, Stacey G. LIK1, a CERK1-interacting kinase, regulates plant immune responses in *Arabidopsis*. PloS one. 2014;9: e1035854. 10.1371/journal.pone.0102245 PMC4103824 25036661

[42] Bourdais G, Burdiak Pl, Gauthier A, Nitsch L, Salojärvi J, Rayapuram C, et al Large-scale phenomics identifies primary and fine-tuning roles for CRKs in responses related to oxidative stress. PLoS Genet. 2015;11: e1005373 10.1371/journal.pgen.1005373 26197346 PMC4511522

[43] Nissen KS, Willats WG, Malinovsky FG. Understanding CrRLK1L Function: Cell Walls and Growth Control. Trends Plant Sci. 2016. 10.1016/j.tplants.2015.12.004 26778775

[44] Stracke S, Kistner C, Yoshida S, Mulder L, Sato S, Kaneko T, et al A plant receptor-like kinase required for both bacterial and fungal symbiosis. Nature. 2002;417: 959–962. 12087405 10.1038/nature00841

[45] Antolín-Llovera M, Ried MK, Parniske M. Cleavage of the SYMBIOSIS RECEPTOR-LIKE KINASE ectodomain promotes complex formation with Nod Factor Receptor 5. Curr Biol. 2014;24: 422–427. 10.1016/j.cub.2013.12.053 24508172

[46] Hok S, Danchin EG, Allasia V, Panabières F, Attard A, Keller H. An *Arabidopsis* (malectin‐like) leucine‐rich repeat receptor‐like kinase contributes to downy mildew disease. Plant, Cell Environ. 2011;34: 1944–1957. 21711359 10.1111/j.1365-3040.2011.02390.x

[47] Yuan Y, Chung JD, Fu X, Johnson VE, Ranjan P, Booth SL, et al Alternative splicing and gene duplication differentially shaped the regulation of isochorismate synthase in *Populus* and *Arabidopsis*. Proc Natl Acad Sci U S A. 2009;106: 22020–22025. 10.1073/pnas.0906869106 19996170 PMC2790362

[48] Hukkanen AT, Kokko HI, Buchala AJ, McDougall GJ, Stewart D, Kärenlampi SO, et al Benzothiadiazole induces the accumulation of phenolics and improves resistance to powdery mildew in strawberries. J Agric Food Chem. 2007;55: 1862–1870. 17279771 10.1021/jf063452p

[49] Wildermuth MC, Dewdney J, Wu G, Ausubel FM. Isochorismate synthase is required to synthesize salicylic acid for plant defence. Nature. 2001;414: 562–565. 11734859 10.1038/35107108

[50] Vlot AC, Dempsey DA, Klessig DF. Salicylic acid, a multifaceted hormone to combat disease. Annu Rev Phytopathol. 2009;47: 177–206. 10.1146/annurev.phyto.050908.135202 19400653

[51] Feys BJ, Wiermer M, Bhat RA, Moisan LJ, Medina-Escobar N, Neu C, et al *Arabidopsis* SENESCENCE-ASSOCIATED GENE101 stabilizes and signals within an ENHANCED DISEASE SUSCEPTIBILITY1 complex in plant innate immunity. Plant Cell. 2005;17: 2601–2613. 16040633 10.1105/tpc.105.033910 PMC1197438

[52] Shah J, Zeier J. Long-distance communication and signal amplification in systemic acquired resistance. Front Plant Sci. 2013;4. 10.3389/fpls.2013.00030 PMC3579191 23440336

[53] Lorenzo O, Piqueras R, Sanchez-Serrano JJ, Solano R. ETHYLENE RESPONSE FACTOR1 integrates signals from ethylene and jasmonate pathways in plant defense. Plant Cell. 2003;15: 165–178. 12509529 10.1105/tpc.007468 PMC143489

[54] Merchante C, Alonso JM, Stepanova AN. Ethylene signaling: simple ligand, complex regulation. Curr Opin Plant Biol. 2013;16: 554–560. 10.1016/j.pbi.2013.08.001 24012247

[55] Yan J, Zhang C, Gu M, Bai Z, Zhang W, Qi T, et al The *Arabidopsis* CORONATINE INSENSITIVE1 protein is a jasmonate receptor. Plant Cell. 2009;21: 2220–2236. 10.1105/tpc.109.065730 19717617 PMC2751961

[56] Saubeau G, Perrin F, Marnet N, Andrivon D, Val F. Hormone signalling pathways are differentially involved in quantitative resistance of potato to *Phytophthora infestans*. Plant Pathol. 2015: 10.1111/ppa.12420

[57] Caarls L, Pieterse CM, Van Wees SC. How salicylic acid takes transcriptional control over jasmonic acid signaling. Front Plant Sci. 2015;6: 10.3389/flps.2015.00170 PMC4373269 25859250

[58] Zheng Z, Qamar SA, Chen Z, Mengiste T. *Arabidopsis* WRKY33 transcription factor is required for resistance to necrotrophic fungal pathogens. Plant J. 2006;48: 592–605. 17059405 10.1111/j.1365-313X.2006.02901.x

[59] Pandey SP, Roccaro M, Schön M, Logemann E, Somssich IE. Transcriptional reprogramming regulated by WRKY18 and WRKY40 facilitates powdery mildew infection of *Arabidopsis*. Plant J. 2010;64: 912–923. 10.1111/j.1365-313X.2010.04387.x 21143673

[60] Agarwal P, Agarwal PK. Pathogenesis related-10 proteins are small, structurally similar but with diverse role in stress signaling. Mol Biol Rep. 2014;41: 599–611. 10.1007/s11033-013-2897-4 24343423

[61] Casado-Díaz A, Encinas-Villarejo S, Santos BDL, Schilirò E, Yubero-Serrano E-, Amil-Ruíz F, et al Analysis of strawberry genes differentially expressed in response to *Colletotrichum* infection. Physiol Plant. 2006;128: 633–650.

[62] Guidarelli M, Carbone F, Mourgues F, Perrotta G, Rosati C, Bertolini P, et al *Colletotrichum acutatum* interactions with unripe and ripe strawberry fruits and differential responses at histological and transcriptional levels. Plant Pathol. 2011;60: 685–697.

[63] Fang X, Jost R, Finnegan PM, Barbetti MJ. Comparative proteome analysis of the strawberry-*Fusarium oxysporum* f. sp. *fragariae* pathosystem reveals early activation of defense responses as a crucial determinant of host resistance. J Proteome Res. 2013;12: 1772–1788. 10.1021/pr301117a 23495785

[64] González G, Fuentes L, Moya-León MA, Sandoval C, Herrera R. Characterization of two PR genes from *Fragaria chiloensis* in response to *Botrytis cinerea* infection: A comparison with *Fragaria* x *ananassa*. Physiol Mol Plant Pathol. 2013;82: 73–80.

[65] Jambagi S, Dunwell J. Global Transcriptome Analysis and Identification of Differentially Expressed Genes after Infection of *Fragaria vesca* with Powdery Mildew (*Podosphaera aphanis*). Transcr Open Access. 2015;2: 2- 10.4172/2329-8936.1000106

[66] Koistinen KM, Soininen P, Venäläinen TA, Häyrinen J, Laatikainen R, Peräkylä M, et al Birch PR-10c interacts with several biologically important ligands. Phytochemistry. 2005;66: 2524–2533. 16246382 10.1016/j.phytochem.2005.09.007

[67] Muñoz C, Hoffmann T, Escobar NM, Ludemann F, Botella MA, Valpuesta V, et al The strawberry fruit Fra a allergen functions in flavonoid biosynthesis. Mol Plant. 2010;3: 113–124. 10.1093/mp/ssp087 19969523

[68] Casañal A, Zander U, Muñoz C, Dupeux F, Luque I, Botella MA, et al The strawberry pathogenesis-related 10 (PR-10) Fra a proteins control flavonoid biosynthesis by binding to metabolic intermediates. J Biol Chem. 2013;288: 35322–35332. 10.1074/jbc.M113.501528 24133217 PMC3853281

[69] Breitfellner F, Solar S, Sontag G. Effect of gamma irradiation on flavonoids in strawberries. Eur Food Res Technol. 2002;215: 28–31.

[70] Wulf J, Rühmann S, Rego I, Puhl I, Treutter D, Zude M. Nondestructive application of laser-induced fluorescence spectroscopy for quantitative analyses of phenolic compounds in strawberry fruits (*Fragaria* x *ananassa*). J Agric Food Chem. 2008;56: 2875–2882. 10.1021/jf072495i 18416555

[71] Kårlund A, Salminen J, Koskinen P, Ahern JR, Karonen M, Tiilikkala K, et al Polyphenols in strawberry (*Fragaria* × *ananassa*) leaves induced by plant activators. J Agric Food Chem. 2014;62: 4592–4600. 10.1021/jf405589f 24819677

[72] Yamamoto M, Nakatsuka S, Otani H, Kohmoto K, Nishimura S. (+)-Catechin acts as an infection-inhibiting factor in strawberry leaf. Phytopathology. 2000;90: 595–600. 10.1094/PHYTO.2000.90.6.595 18944538

[73] Puhl I, Treutter D. Ontogenetic variation of catechin biosynthesis as basis for infection and quiescence of *Botrytis cinerea* in developing strawberry fruits. J Plant Dis Prot. 2008;115: 247–251.

[74] Kanehisa M, Goto S. KEGG: kyoto encyclopedia of genes and genomes. Nucleic Acids Res. 2000;28: 27–30. 10592173 10.1093/nar/28.1.27 PMC102409

[75] Vranová E, Coman D, Gruissem W. Structure and dynamics of the isoprenoid pathway network. Mol Plant. 2012;5: 318–333. 10.1093/mp/sss015 22442388

[76] Hirai N, Sugie M, Wada M, Lahlou EH, Kamo T, Yoshida R, et al Triterpene phytoalexins from strawberry fruit. Biosci Biotechnol Biochem. 2000;64: 1707–1712. 10993160 10.1271/bbb.64.1707

[77] Thimmappa R, Geisler K, Louveau T, O'Maille P, Osbourn A. Triterpene biosynthesis in plants. Annu Rev Plant Biol. 2014;65: 225–257. 10.1146/annurev-arplant-050312-120229 24498976

[78] Hanhineva K, Rogachev I, Kokko H, Mintz-Oron S, Venger I, Kärenlampi S, et al Non-targeted analysis of spatial metabolite composition in strawberry (*Fragaria* × *ananassa*) flowers. Phytochemistry. 2008;69: 2463–2481. 10.1016/j.phytochem.2008.07.009 18774147

[79] Augustin JM, Kuzina V, Andersen SB, Bak S. Molecular activities, biosynthesis and evolution of triterpenoid saponins. Phytochemistry. 2011;72: 435–457. 10.1016/j.phytochem.2011.01.015 21333312

[80] Nomura T, Sato T, Bishop GJ, Kamiya Y, Takatsuto S, Yokota T. Accumulation of 6-deoxocathasterone and 6-deoxocastasterone in *Arabidopsis*, pea and tomato is suggestive of common rate-limiting steps in brassinosteroid biosynthesis. Phytochemistry. 2001;57: 171–178. 11382232 10.1016/s0031-9422(00)00440-4

[81] Lozano-Durán R, Zipfel C. Trade-off between growth and immunity: role of brassinosteroids. Trends Plant Sci. 2015;20: 12–19. 10.1016/j.tplants.2014.09.003 25278266

[82] Albrecht C, Boutrot F, Segonzac C, Schwessinger B, Gimenez-Ibanez S, Chinchilla D, et al Brassinosteroids inhibit pathogen-associated molecular pattern-triggered immune signaling independent of the receptor kinase BAK1. Proc Natl Acad Sci U S A. 2012;109: 303–308. 10.1073/pnas.1109921108 22087006 PMC3252947

[83] Belkhadir Y, Jaillais Y, Epple P, Balsemao-Pires E, Dangl JL, Chory J. Brassinosteroids modulate the efficiency of plant immune responses to microbe-associated molecular patterns. Proc Natl Acad Sci U S A. 2012;109: 297–302. 10.1073/pnas.1112840108 22087001 PMC3252953

[84] De Vleesschauwer D, Van Buyten E, Satoh K, Balidion J, Mauleon R, Choi IR, et al Brassinosteroids antagonize gibberellin- and salicylate-mediated root immunity in rice. Plant Physiol. 2012;158: 1833–1846. 10.1104/pp.112.193672 22353574 PMC3320189

[85] Liu H, Xie W, Zhang L, Valpuesta V, Ye Z, Gao Q, et al Auxin biosynthesis by the YUCCA6 flavin monooxygenase gene in woodland strawberry. J Integr Plant Biol. 2014;56: 350–363. 10.1111/jipb.12150 24373096

[86] Zhao Y, Christensen SK, Fankhauser C, Cashman JR, Cohen JD, Weigel D, et al A role for flavin monooxygenase-like enzymes in auxin biosynthesis. Science. 2001;291: 306–309. 11209081 10.1126/science.291.5502.306

[87] Mashiguchi K, Tanaka K, Sakai T, Sugawara S, Kawaide H, Natsume M, et al The main auxin biosynthesis pathway in Arabidopsis. Proc Natl Acad Sci U S A. 2011;108: 18512–18517. 10.1073/pnas.1108434108 22025724 PMC3215075

[88] Kazan K, Manners JM. Linking development to defense: auxin in plant–pathogen interactions. Trends Plant Sci. 2009;14: 373–382. 10.1016/j.tplants.2009.04.005 19559643

[89] Denancé N, Ranocha P, Oria N, Barlet X, Rivière M-, Yadeta KA, et al Arabidopsis *wat1* (*walls are thin1*)-mediated resistance to the bacterial vascular pathogen, *Ralstonia solanacearum*, is accompanied by cross-regulation of salicylic acid and tryptophan metabolism. Plant J. 2013;73: 225–239. 10.1111/tpj.12027 22978675

[90] Ranocha P, Dima O, Nagy R, Felten J, Corratgé-Faillie C, Novák O, et al Arabidopsis WAT1 is a vacuolar auxin transport facilitator required for auxin homoeostasis. Nat Commun. 2013;4: 10.1038/ncomms3625 PMC3826630 24129639

[91] Ding X, Cao Y, Huang L, Zhao J, Xu C, Li X, et al Activation of the indole-3-acetic acid-amido synthetase GH3-8 suppresses expansin expression and promotes salicylate- and jasmonate-independent basal immunity in rice. Plant Cell. 2008;20: 228–240. 10.1105/tpc.107.055657 18192436 PMC2254934

[92] Mutka AM, Fawley S, Tsao T, Kunkel BN. Auxin promotes susceptibility to *Pseudomonas syringae* via a mechanism independent of suppression of salicylic acid‐mediated defenses. Plant J. 2013;74: 746–754. 10.1111/tpj.12157 23521356

[93] Eshraghi L, Anderson JP, Aryamanesh N, McComb JA, Shearer B, Hardy GS. Suppression of the auxin response pathway enhances susceptibility to *Phytophthora cinnamomi* while phosphite-mediated resistance stimulates the auxin signalling pathway. BMC Plant Biol. 2014;14: 68-2229-14-68. 10.1186/1471-2229-14-68 PMC3999932 24649892

[94] Zhong R, Richardson EA, Ye ZH. The MYB46 transcription factor is a direct target of SND1 and regulates secondary wall biosynthesis in *Arabidopsis*. Plant Cell. 2007;19: 2776–2792. 17890373 10.1105/tpc.107.053678 PMC2048704

[95] Zhou J, Zhong R, Ye Z. *Arabidopsis* NAC domain proteins, VND1 to VND5, are transcriptional regulators of secondary wall biosynthesis in vessels. PloS one. 2014;9: e105726 10.1371/journal.pone.0105726 25148240 PMC4141820

[96] Hussey SG, Mizrachi E, Spokevicius AV, Bossinger G, Berger DK, Myburg AA. SND2, a NAC transcription factor gene, regulates genes involved in secondary cell wall development in *Arabidopsis* fibres and increases fibre cell area in *Eucalyptus*. BMC Plant Biol. 2011;11: 173-2229-11-173. 10.1186/1471-2229-11-173 PMC3289092 22133261

[97] MacMillan CP, Mansfield SD, Stachurski ZH, Evans R, Southerton SG. Fasciclin‐like arabinogalactan proteins: specialization for stem biomechanics and cell wall architecture in *Arabidopsis* and *Eucalyptus*. Plant J. 2010;62: 689–703. 10.1111/j.1365-313X.2010.04181.x 20202165

[98] Mewalal R, Mizrachi E, Mansfield SD, Myburg AA. Cell wall-related proteins of unknown function: missing links in plant cell wall development. Plant Cell Physiol. 2014;55: 1031–1043. 10.1093/pcp/pcu050 24683037

[99] Miedes E, Vanholme R, Boerjan W, Molina A. The role of the secondary cell wall in plant resistance to pathogens. Front Plant Sci. 2015: 78. 25161657 10.3389/fpls.2014.00358 PMC4122179

[100] Caño‐Delgado A, Penfield S, Smith C, Catley M, Bevan M. Reduced cellulose synthesis invokes lignification and defense responses in *Arabidopsis thaliana*. Plant J. 2003;34: 351–362. 12713541 10.1046/j.1365-313x.2003.01729.x

