# Supplementary figures and images for: Reprogramming of Strawberry (Fragaria vesca) Root Transcriptome in Response to Phytophthora cactorum
Source: PLoS One. 2016 Aug 12;11(8):e0161078. doi: 10.1371/journal.pone.0161078 (PMC4982697; doi:10.1371/journal.pone.0161078)

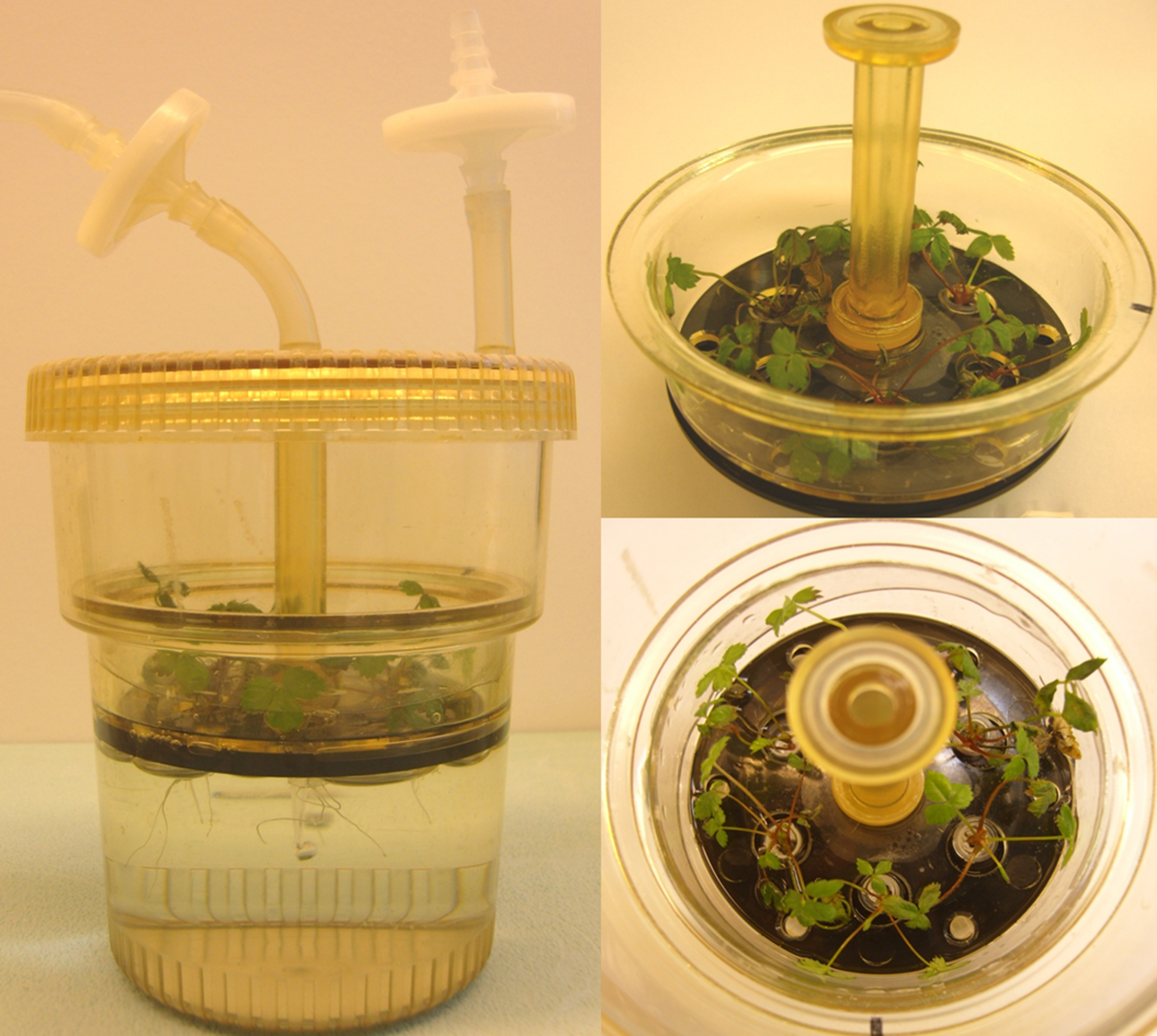

Supplement: S1 Fig — The plants were grown and inoculated in the aerated hydroponic cultures in modified RITA® containers (VITROPIC, Saint-Mathieu-de-Tréviers, France). (TIF) [file pone.0161078.s001.tif]

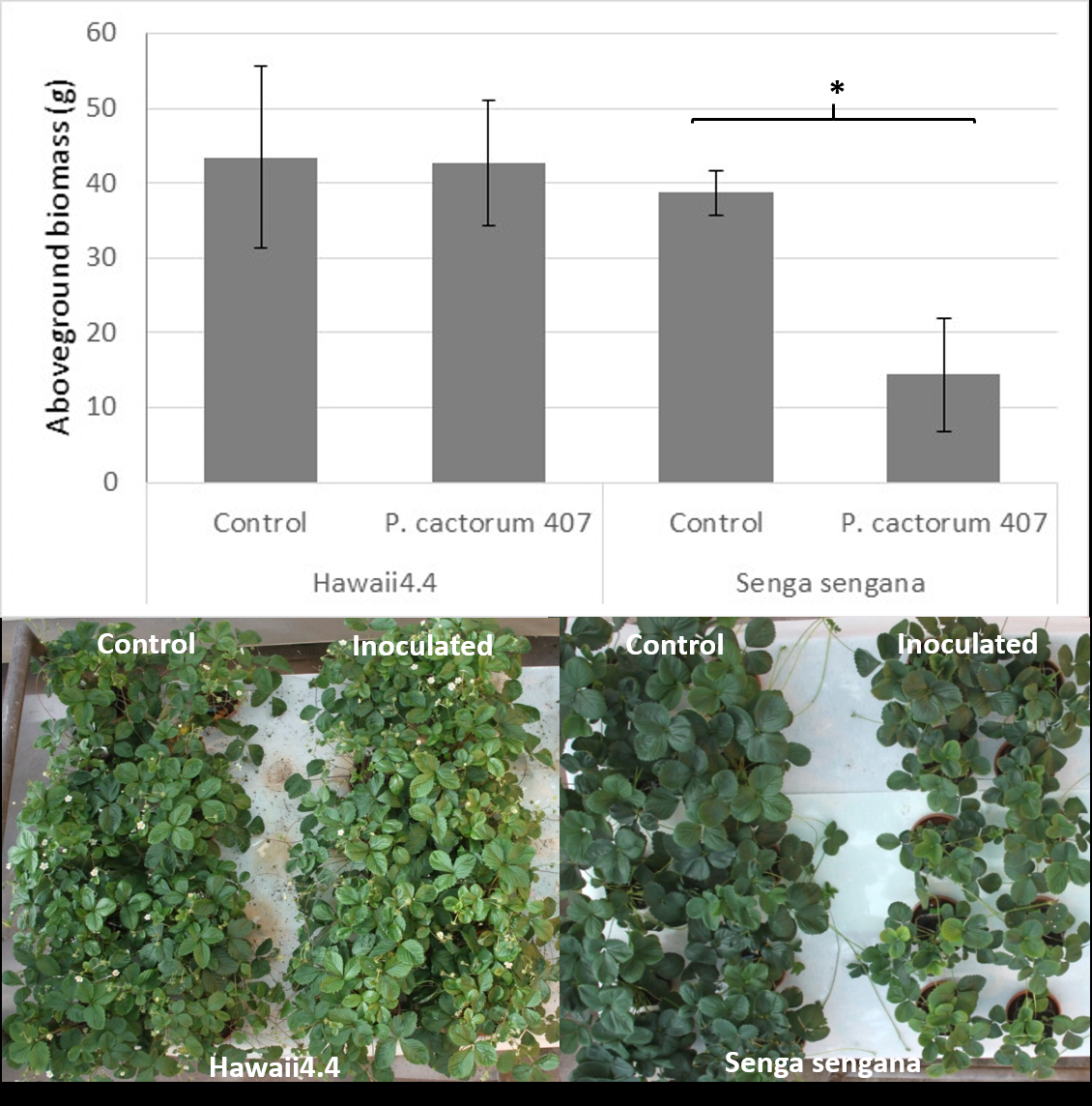

Supplement: S2 Fig — Micropropagated plants were grown in pots in peat-sand mixture (3:1) for three weeks and inoculated four times with 5 ml of zoospore suspension 21, 29, 32, and 43 days after potting. The zoospore concentrations were 47 500, 130 000, 49 000, 41 000 zoospores /ml, respectively. Biomasses were measured 57 days after first inoculation. P. cactorum isolate 407 did not reduce the growth of Hawaii 4.4 genotype, but inoculated Senga Sengana plants were severely stunted compared to the controls (p = 0.000001). The means are derived from ten replicates. (TIF) [file pone.0161078.s002.tif]

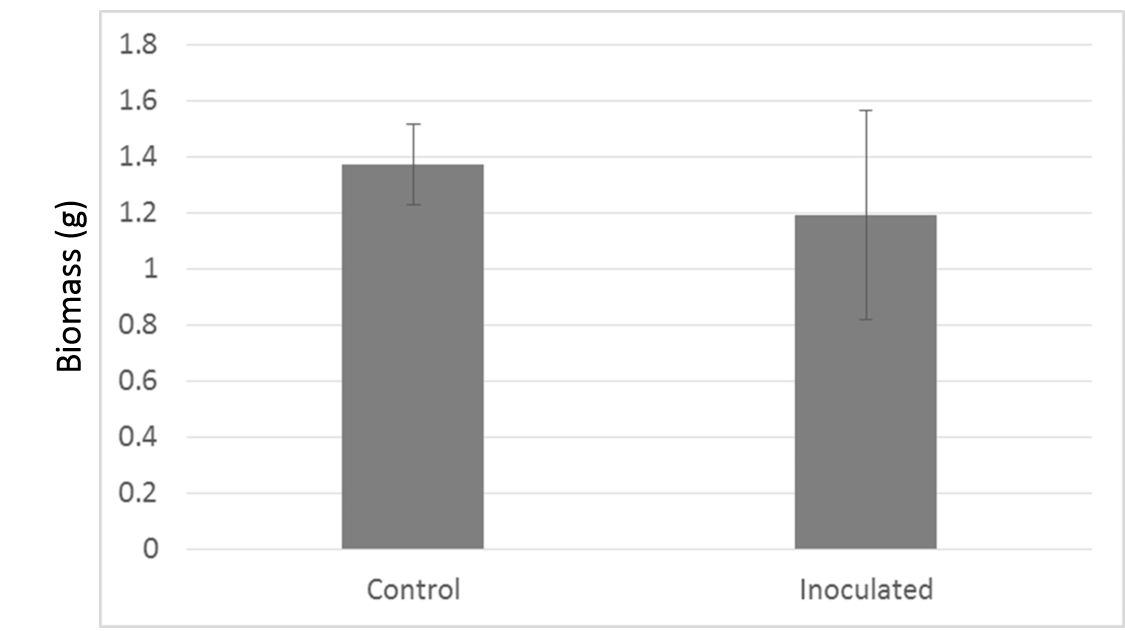

Supplement: S3 Fig — Micropropagated plants were grown in hydroponic cultures for 32 days, and then inoculated by dipping the roots in zoospore suspension (500 zoospores/ml) for two hours. Biomasses were measured 8 weeks after inoculation. P. cactorum inoculation did not cause significant reduction in biomass of Hawaii 4.4 plants. (TIF) [file pone.0161078.s003.tif]

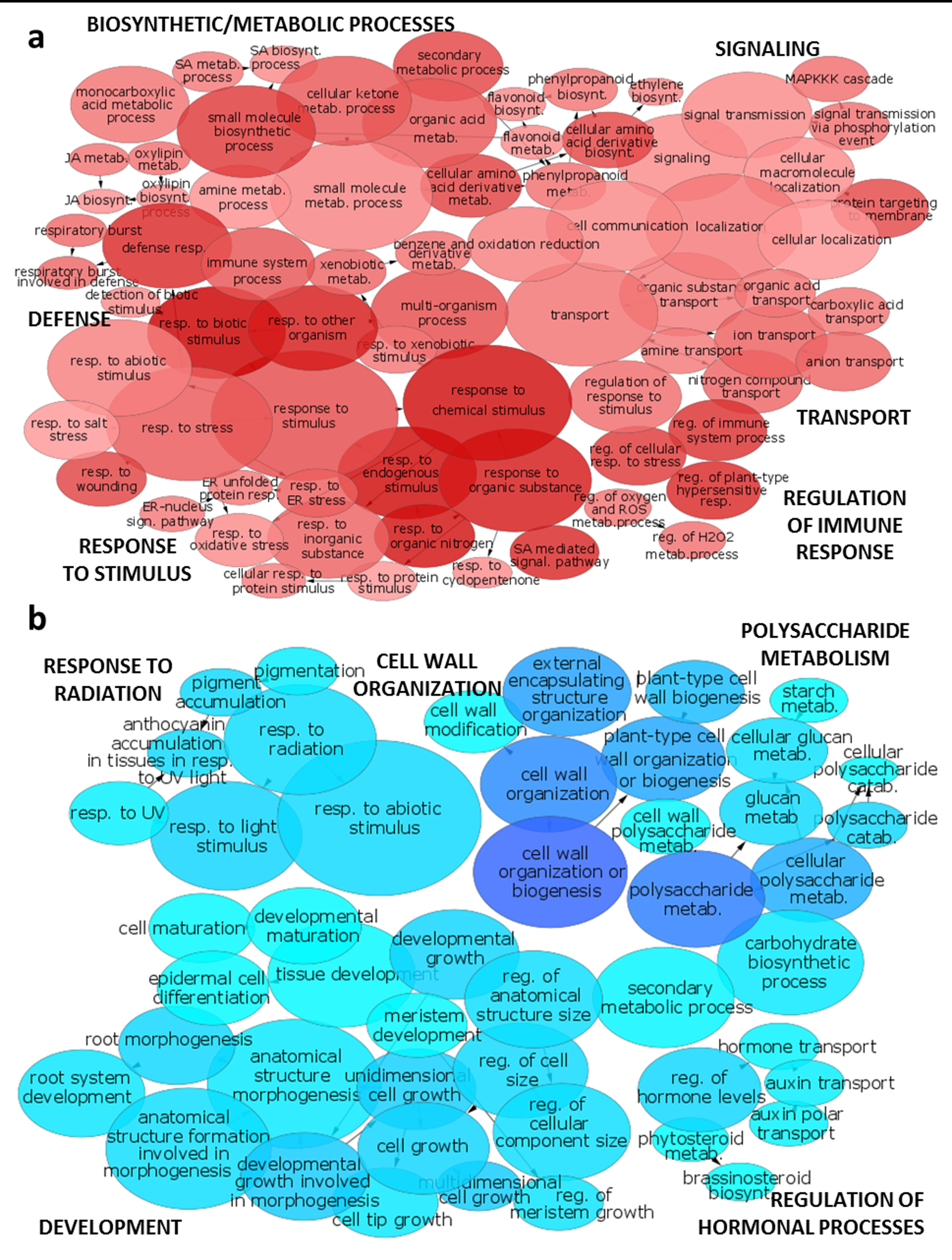

Supplement: S4 Fig — (A) up-regulated, (B) down-regulated (adjusted p-value < 1E-15). The most redundant GO terms based on REVIGO analysis were removed to improve clarity. Cutoff value for similarity of 0.7 was used for up-regulated and of 0.9 for the down-regulated processes. The intensity of the color indicates the degree of significance and the node size is proportional to the number of the genes assigned to each GO term in the tested gene set. (TIF) [file pone.0161078.s004.tif]

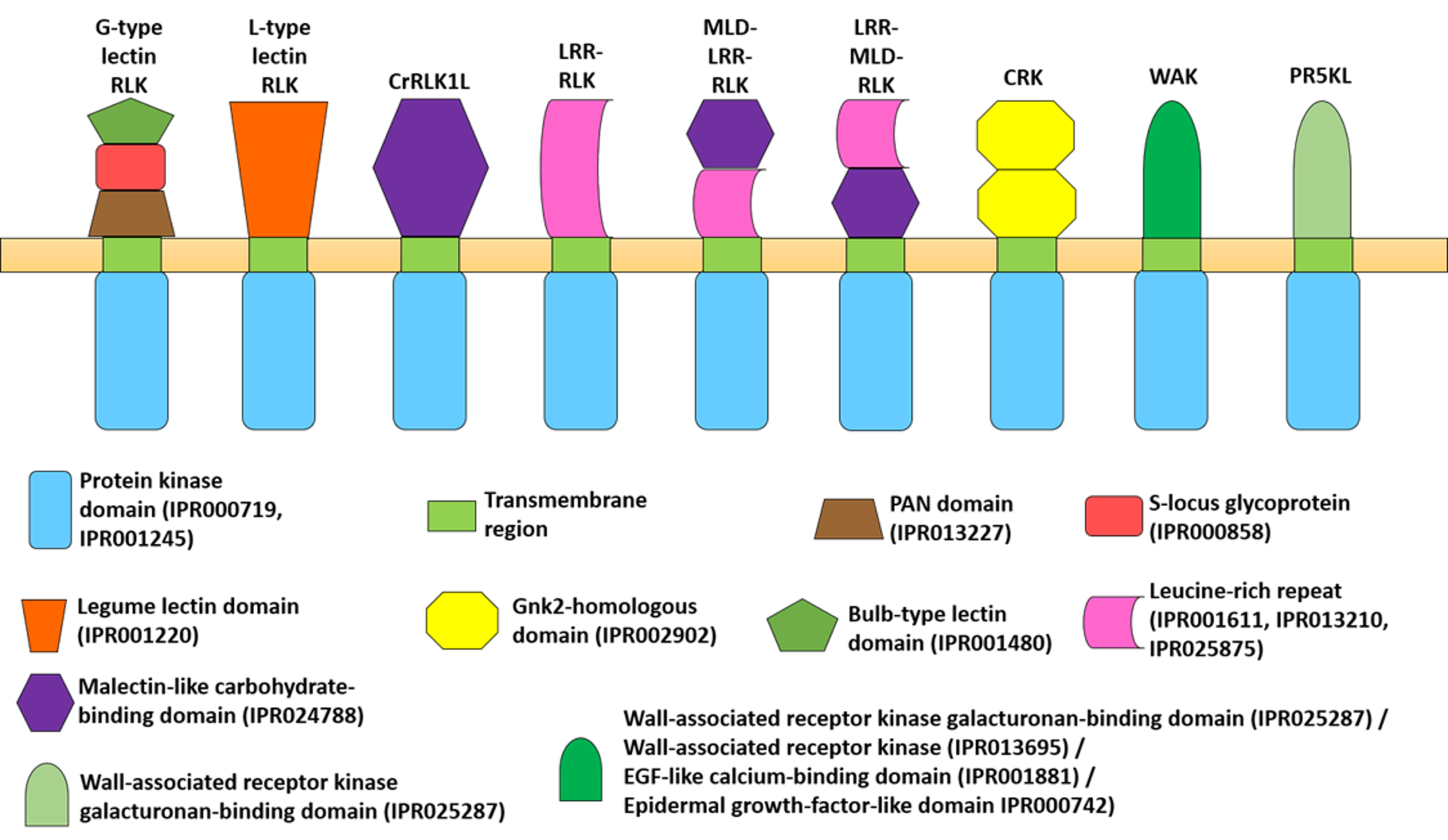

Supplement: S5 Fig — (TIF) [file pone.0161078.s005.tif]

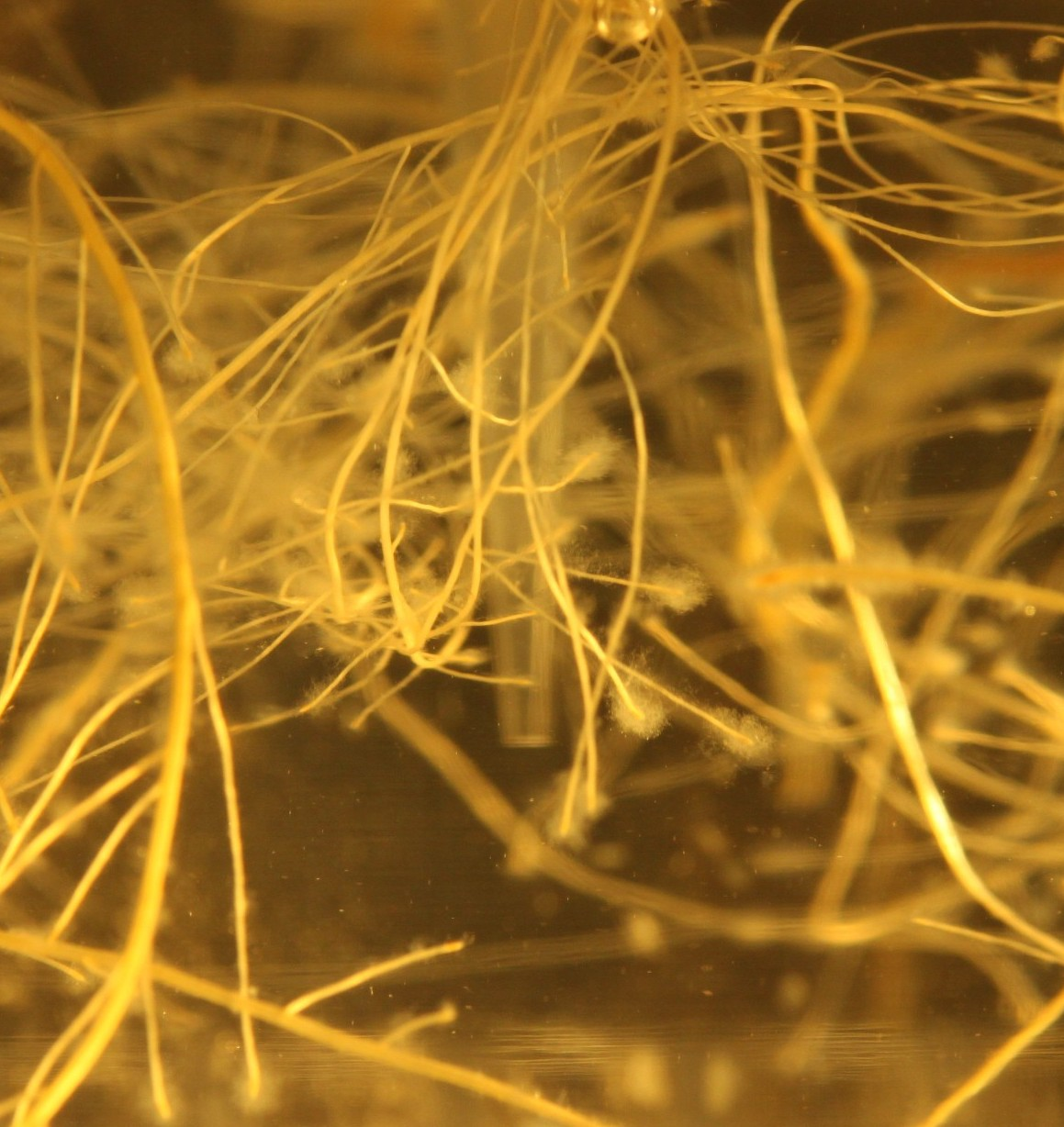

Supplement: S6 Fig — (TIF) [file pone.0161078.s006.tif]
